# Gliovirin-like Alkaloids with Spirocyclic Skeletons from the Mangrove Endophytic Fungus *Penicillium janthinellum* HDN13-309

**DOI:** 10.3390/md24080258

**Published:** 2026-07-26

**Authors:** Haotian Wang, Runyu Wu, Lanying Li, Huimin Feng, Yixuan Zhang, Yajuan Cong, Dehai Li, Hao Liu, Meilin Zhu

**Affiliations:** 1 School of Pharmacy, Bengbu Medical University, Bengbu 233030, China; haotian@bbmu.edu.cn (H.W.);; 2 School of Medicine and Pharmacy, Ocean University of China, Qingdao 266003, China

**Keywords:** natural product, mangrove endophytic fungus, ETP, penicisulfuranols, anticancer

## Abstract

To fully explore the metabolites of the mangrove endophytic fungus *Penicillium janthinellum* HDN13-309, which can produce Epipolythiodioxopiperazine (ETP) compounds with noteworthy anti-tumor activities, HPLC-MS analysis was applied, and five new gliovirin-like alkaloids, penicisulfuranols G–K (**1**–**5**), were target-directed acquired. Furthermore, penicisulfuranols G (**1**) and H (**2**) were distinguished by a double bond between C-5/C-6 and ortho-hydroxylation of C-7/C-8 on the 1,2-oxazadecaline moiety. Their structures, including absolute configurations, were elucidated based on NMR spectroscopy, ECD measurements, and high-resolution mass spectrometry, as well as by comparison with the literature. Penicisulfuranols I–K (**3**–**5**) showed promising cytotoxicity against four tumor cell lines (HCT116, HeLa, HL-60, and K562) with IC_50_ values ranging from 0.1 to 9.9 μM.

## 1. Introduction

Epipolythiodioxopiperazines (ETPs) are a class of bioactive fungal secondary metabolites featuring a central 2,5-diketopiperazine (DKP) moiety and a bridging polysulfide [1,2]. This intramolecular disulfide bridge usually exists on the *α*-positions of the two amino acid residues that form DKP. It has been reported that the disulfide bond in ETPs is stabilized by two *n*→π* interactions, which in turn affects their biological activity [3]. Meanwhile, some atypical ETPs have been identified; gliovirin-like compounds are ETP derivatives possessing sulfide bridges between *α*- and *β*-carbons, and an uncommon 1,2-oxazadecaline moiety [4], with some featuring a spirofuran skeleton such as peniciadametizine A [5]. These ETPs have been synthesized by medicinal chemists because of their peculiar structural complexity and noteworthy bioactivities. To date, the total synthesis of ETPs remains challenging, and only one *α*,*β*-disulfide bridge ETP derivative has been accomplished [6,7,8,9,10]. Recent studies have revealed that the characteristic *α*,*β*-disulfide bridge in ETPs is generated through sequential *α*,*α*-disulfide formation and specialized oxygenase-mediated disulfide migration, providing key mechanistic insights into the biosynthesis and structural diversification of this unique ETP subclass [11,12]. Meanwhile, investigations into tailoring enzymes involved in ETP biosynthesis in *Trichoderma hypoxylon* have uncovered a hidden reservoir of structurally diverse ETP alkaloids in fungi, highlighting biosynthetic engineering as a promising strategy for accessing novel ETP analogs [13,14].

As privileged natural sources, mangrove endophytic fungi thrive in high salinity and high amounts of organic matter that trigger unique biosynthetic pathways to produce structurally rare metabolites with promising anticancer potential [15]. In our previous study, a mangrove endophytic fungus, *Penicillium janthinellum* HDN13-309, was found to produce a series of spiro-fused ETPs including penisuloxazin A, penicisulfuranols A–F, and penispirozines A–H [16,17,18]. Among these, penicisulfuranols A–F, known as the gliovirin-like alkaloids, are highly unusual as they contain an irregular *α*, *β*-sulfide bridge; a 1, 2-oxazine moiety; and a spiro-fused benzofuranpiperazinedione ring system. Beyond their peculiar structures, these compounds are important because they exhibited strong cytotoxic activities, and mechanism studies showed that penicisulfuranol A was a novel C-terminal inhibitor of Hsp90, a strategy considered a promising approach for cancer treatment that warrants further investigation for drug development [18,19,20].

**Figure 1:**
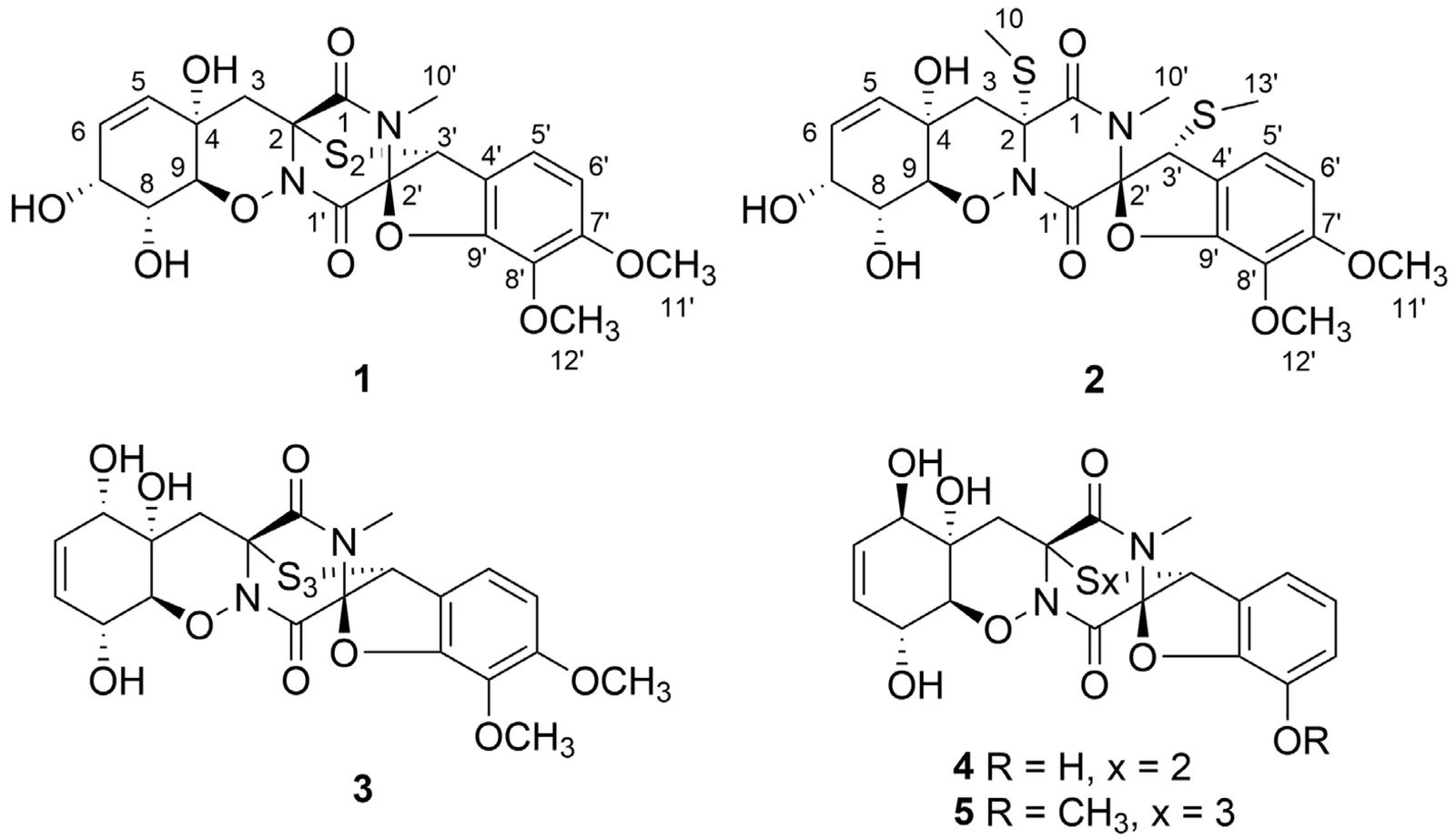
Structures of compounds **1**–**5**.

Considering the intricacies of natural products and high-resolution performance of MS, MS data have been utilized for preliminary identification of the crude extract, as well as for elucidating novel compounds [21]. In an attempt to identify more ETP derivatives, HPLC-MS-guided separation of the EtOAc extract of *Penicillium janthinellum* HDN13-309 was applied, and the results led to the isolation of five new gliovirin-like ETPs, named penicisulfuranols G–K (**1**–**5**), as shown in Figure 1. Notably, penicisulfuranols G (**1**) and H (**2**) represent unprecedented *α*,*β*-disulfide-containing gliovirin analogs featuring a rearranged spirofuran skeleton accompanied by an unusual migration of the olefinic bond from the C-6/C-7 position to C-5/C-6, thereby expanding the structural diversity of ETP alkaloids. Herein, the isolation, structural elucidation, and bioactivity evaluation of compounds **1**–**5** were elucidated.

## 2. Results

HPLC-MS analysis (positive ion) of the crude extract from the strain revealed the presence of penigainamide A at the ion peak of *m*/*z* 585.2 [M + H]^+^ and several potential ETP-type compounds which were uncharacterized previously (Figure S1). The ion peak of *m*/*z* 533.1 showed that compound **1** was short by one sulfur atom compared to penicisulfuranol C^16^, and that of *m*/*z* 565.0 (compound **3**) indicated a potential isomer of penicisulfuranol C. The ion peaks of *m*/*z* 467.0 [M + H]^+^ and 489.0 [M + Na]^+^ suggest the possibility that compound **4** might differ from penicisulfuranols by methoxy or hydroxyl groups. Through tracking target compounds, five new gliovirin analogs (**1**–**5**) were determined from the EtOAc extract of the strain.

**Figure 2:**
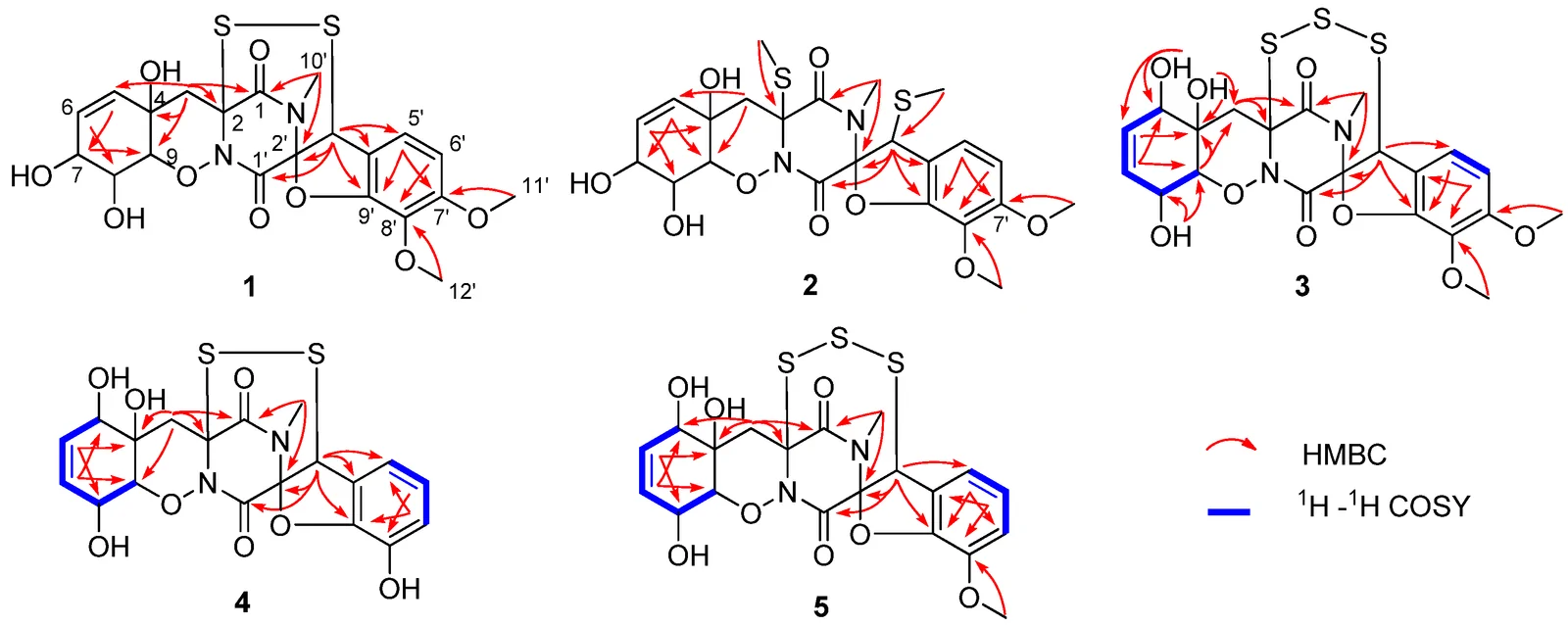
Key COSY and HMBC correlations of compounds **1**–**5**.

Penicisulfuranol G (**1**) was isolated as a pale yellow powder with the molecular formula C_21_H_22_N_2_O_9_S_2_ determined from a HRESIMS peak at *m*/*z* 511.0842 [M + H]^+^. A thorough analysis of the ^1^H and ^13^C NMR data of **1** with DEPT and HSQC techniques revealed the existence of three methyls (with two of them oxygenated), four sp^2^-methines, four sp^3^-methines (with three of them oxygenated), one sp^3^-methylene, and nine quaternary carbons, which are similarly consistent with those of penicisulfuranol B^16^. Detailed analysis of the key HMBC correlations from H_2_-3 to C-4/C-5/C-9, from H-5 to C-7/C-9, from H-6 to C-8, and from H-7 to C-9 established the cyclohexene ring with the double bond placed at C-5 and C-6, with the hydroxyl group located at C-7. The spiro-fused benzofuranpiperazinedione moiety was established by HMBC correlations from H-3′ to C-1′/C-2′/C-4′/ C-5′/C-9′, from H-5′ to C-7′/C-9′, from H-6′ to C-8′ and from the 11′-OCH_3_ and 12′-OCH_3_ to C-7′ and C-8′, respectively. Thus, the planar structure of compound **1** was established as shown in Figure 2, and the *∆*^5, 6^ and 7-OH distinguished **1** from other known penicisulfuranols [22,23].

**Figure 3:**
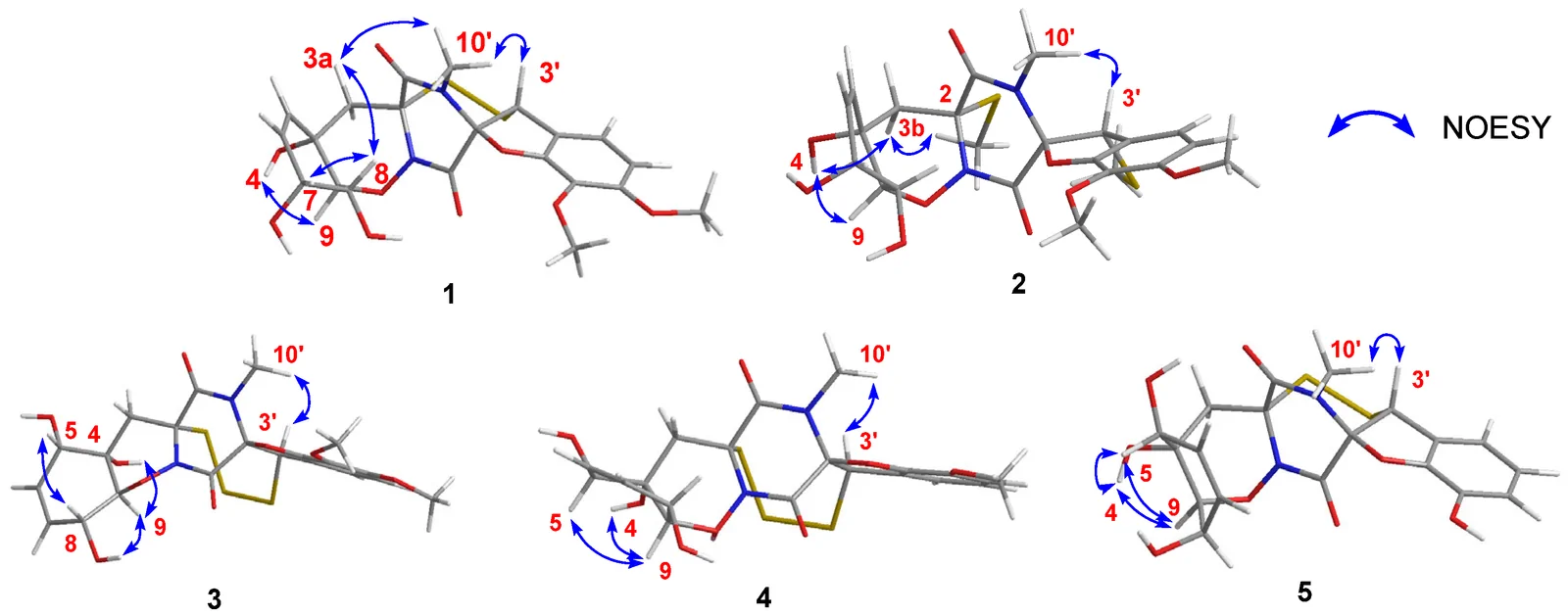
Key NOESY correlations of compounds **1**–**5**.

**Figure 4:**
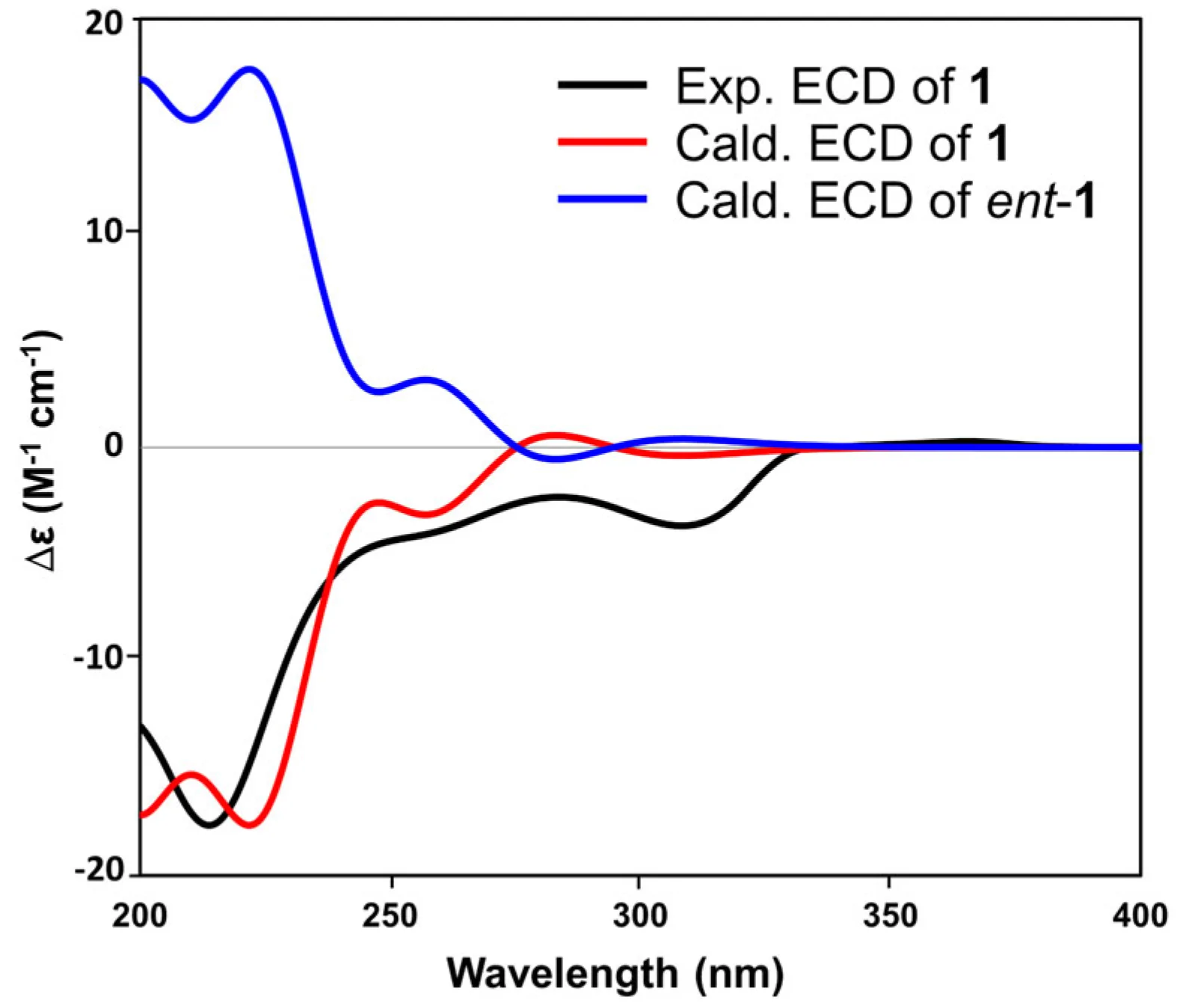
B3LYP/6-31+G(d)-calculated ECD spectra of **1** (red line) and its enantiomer (blue line) and the experimental one of **1** (black line) (*σ* = 0.27 eV for **1**).

The relative configuration of penicisulfuranol G (**1**) was determined as 2R*, 4S*, 7R*, 8R*, 9S*, 2′R*, and 3′R* based on the NOESY correlations and coupling constants, as well as comparing the NMR data of **1** with those of pretrichodermamides D and E (a pair of diastereoisomers with a different absolute configuration of C-7) [23], as shown in Figure 3. The NOESY cross-peaks of H-7/H-8 and the small coupling constant between them (4.4 Hz) indicated that H-7 and H-8 were cofacial on the cyclohexene ring. This assignment was consistent with the NMR data of pretrichodermamide D (which possesses a *cis* H-7/H-8 configuration, *δ*_C_ 65.8 and 66.2 for C-7 and C-8) and clearly differs from those of pretrichodermamide E (*trans*, *δ*_C_ 72.2 and 71.0 for C-7 and C-8) [23]. The NOESY correlation of 4-OH/H-9 and the large coupling constant of H-8 and H-9 (9.9 Hz) suggested that 4-OH and H-9 oriented to the other side of the hexane ring. The relative configurations of C-2′ and C-3′ were assigned as that of known penicisulfuranol B according to the key NOESY correlations of H-3a/H_3_-10′/H-8/H-3′. The absolute configuration of **1** was determined by time-dependent density functional theory (TDDFT) ECD calculations. Five conformers accounting for >5% Boltzmann population were obtained (Figure S2). The ECD spectrum of each conformer was calculated at the same basis set, and the combination results showed good agreement with the experimental ones (Figure 4), which indicated the 2*R*, 4*S*, 7*R*, 8*R*, 9*S*, 2′*R*, and 3′*R* absolute configuration of **1**.

Penicisulfuranol H (**2**) was isolated with molecular formula C_23_H_28_N_2_O_9_S_2_ based on the HRESIMS ion peak at *m*/*z* 563.1127 [M + Na]^+^. Analysis of the 1D NMR indicated that compound **2** showed striking resemblance to compound **1**, except for the presence of two S-methyls (*δ*_H_ 2.22, 2-SMe; *δ*_H_ 2.06, 3′-SMe). The key HMBC correlations from H_3_-10 to C-2 and from H_3_-13′ to C-3′ suggested that the S-methyl groups were located on C-2 and C-3′, respectively. The absolute configuration of **2** was deduced to be that of **1** based on NOE data, coupling constants, and biogenetic consideration.

Penicisulfuranol I (**3**) was isolated as a pale yellow solid with the molecular formula C_21_H_22_N_2_O_9_S_3_ determined from a HRESIMS peak at *m*/*z* 565.0388 [M + Na]^+^. A thorough analysis of the ^1^H and ^13^C NMR data of **3** with DEPT and HSQC techniques revealed the presence of four sp^2^-methines, four sp^3^-methines (with three of them oxygenated), one sp^3^-methylene, and three methyls (with two of them oxygenated), together with nine quaternary carbons. The general features of the ^1^H and ^13^C NMR spectra of compound **3** resembled those of penicisulfuranol C^16^, an active anti-tumor ETP isolated from the same fungus, except for signals at C-3, C-4, C-5, C-6, C-7, and C-8. The key HMBC correlations from H-3 to C-1, C-2, C-5, and C-9; from H-5 to C-3, C-7, and C-9; from H-8 to C-4 and C-6; and from H-9 to C-3, C-4, and C-5 established the same planar structure with penicisulfuranol C. NOESY correlations from H-9 to 4-OH and 8-OH and from H-5 to H-8, together with the large coupling constants of H-8 and H-9 (7.2 Hz), indicated the *α*-orientation of 5-OH and detected **3** as the C-5-epimer of penicisulfuranol C^16^. According to experimental ECD spectra, the absolute configurations in compound **3** were proposed as 2*R*, 4*S*, 5*S*, 8*R*, 9*S*, 2′*R*, and 3′*R* based on biogenetic relationships with penicisulfuranols A–F, as shown in Figure S4. It is worth noting that the ECD spectrum of compound **3** showed close similarity to that of penicisulfuranol B, despite the different configuration at C-5. This phenomenon can be rationalized by the fact that the spiro-fused benzofuranpiperazinedione moiety serves as the dominant chromophore, while the remote C-5 stereocenter exerts only a minor influence on the overall Cotton effects, as has been observed in other ETP alkaloids (pretrichodermamide C and pretrichodermamide F) [23].

**Figure 5:**
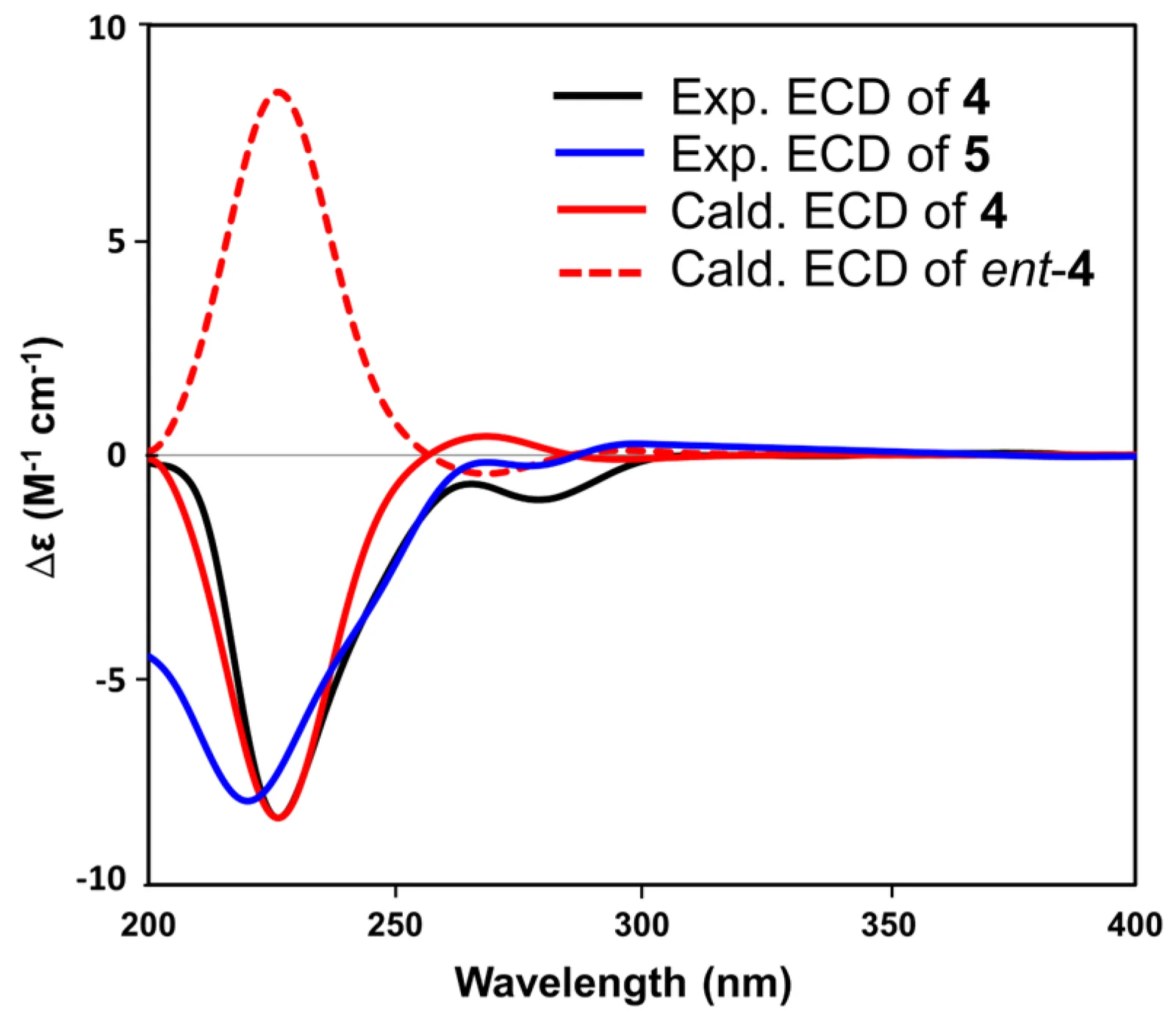
B3LYP/6-31+G(d)-calculated ECD spectra of **4** (red line) and its enantiomer (red dashed line) and the experimental ones of **4** and **5** (blue and black lines) (*σ* = 0.25 eV for **4**).

Penicisulfuranol J (**4**) was obtained as a pale yellow powder, with a chemical molecular formula deduced as C_19_H_18_N_2_O_8_S_2_ according to HRESIMS analysis. Further examination of the NMR data of **4** showed that it shared a similar skeleton to penicisulfuranol B. Nevertheless, slight differences were observed in the signals of the phenyl ring. Comparison of the ^1^H NMR data with penicisulfuranol B revealed the disappearance of two methoxy groups and the existence of an aromatic hydrogen proton (*δ*_H_ 6.83, H-7′) and a phenolic hydroxyl proton (*δ*_H_ 9.9, 8′-OH). Comprehensive analysis of the 2D NMR data of **2** including COSY correlations from H-5′ to H-7′ as well as HMBC correlations from H-7′ to C-5′ and C-9′ and from 8′-OH to C-8′, further confirmed the structural determination of **4**. Evidenced by the NOESY correlations between H-9/4-OH/H-5 and H-3′/Me-10′, as well as the large coupling constants between H-8 and H-9 (7.3 Hz), the relative configuration of compound **4** was suggested to be that of penicisulfuranol B. Finally, based on biosynthetic considerations and the comparison of its experimental ECD spectrum with the calculated ECD spectra, the absolute configuration of **4** was assigned as 2*R*, 4*R*, 5*R*, 8*R*, 9*S*, 2′*R*, and 3′*R* (Figure 5).

Penicisulfuranol K (**5**) was isolated as a pale yellow powder and assigned the molecular formula C_20_H_20_N_2_O_8_S_3_ based on the HRESIMS ion peak at *m*/*z* 535.0272 [M + Na]^+^. Considering the MS data and the chemical shift differences in C-2 and C-3′ between 4 and 5 (*δ*_C_ 69.2 and 51.9 in **4** vs. 77.0 and 65.2 in **5**), it is suggested that compound **5** was the trisulfide analog. In addition, the appearance of signals for the methyl group (*δ*_H_ 3.79, *δ*_C_ 56.2) and HMBC correlations from H_3_-11′ to C-8′ indicated that OH was substituted by a methoxyl in **5**. The similar NOESY correlations, coupling constants, and ECD spectrum indicated that it shared the same relative configurations as **4** (Figure 5).

The cytotoxicity of compounds **1**–**5** was evaluated against HCT116, HeLa, HL-60, and K562 cell lines. As shown in Table 1, compounds **3**–**5** were active against those cells with IC_50_ values ranging from 0.1 to 9.9 μM, while compounds **1** and **2** did not have observable cytotoxic activities against the four tumor cells (IC_50_ > 30 μM).

## 3. Discussion

The HPLC-MS-guided investigation of *Penicillium janthinellum* HDN13-309 expands the chemical profile of gliovirin-like ETPs by adding five new derivatives, penicisulfuranols G–K (**1**–**5**). These findings support the working hypothesis that the strain still contains an underexplored reservoir of ETP alkaloids and reinforce previous evidence that mangrove endophytic fungi exhibit promising potential for producing structurally unusual metabolites.

Penicisulfuranols G–H (**1**–**2**) are especially noteworthy because they represent the rare gliovirin analogs combining a spirocyclic skeleton with migration of the double bond to C-5/C-6 in the 1,2-oxazadecaline moiety. Together with penicisulfuranols I–K (**3**–**5**), these structures demonstrate substantial biosynthetic diversity of the metabolites from the strain. To date, only four compounds (pretrichodermamides D–E, trichodermamides D–E) featuring a *Δ*^5, 6^ isomerization at the oxazadecaline moiety have been identified [22,23]. All these compounds were produced by *Penicillium* sp. fungi, while the biosynthetic basis underlying the formation of these unusual ETP derivatives remains unexplored. In light of recent biosynthetic studies on ETPs from *Trichoderma hypoxylon*, compounds **1** and **2** are proposed to undergo C-6/C-7 dihydroxylation and dehydration, followed by cytochrome P450 monooxygenase-catalyzed epoxidation and spontaneous hydration, leading to the formation of pretrichodermamides D–E and trichodermamides D–E [13,14]. Further experimental validation of this proposed biosynthetic pathway may provide valuable insights into the hidden chemical diversity and biosynthetic diversification of ETPs.

The cytotoxicity results reveal a clear preliminary structure–activity relationship. Compounds **3**–**5** show activities against HCT116, HeLa, HL-60, and K562 cells, whereas **1** and **2** exhibit no significant cytotoxic effects. Thus, activity appears to be determined not simply by the ETP skeleton, but by the precise sulfur bridge, stereochemistry, ring conformation, and aromatic substitution pattern. The inactivity of **1** and **2** suggests that double-bond migration and the associated spirocyclic/conformational rearrangement may disrupt the spatial or electronic features required for cytotoxicity. By contrast, the retained activity of **3**–**5** indicates that C-5 epimerization, phenolic demethylation, and trisulfide formation are tolerated. Furthermore, similar binding patterns have been reported for related analogs PNSA, PEN-A, and HDN-1, which interact with cysteine residues within the C-terminal domain of Hsp90, suggesting that these conserved cysteine residues may serve as potential binding sites for this class of compounds [18,19]. Although the inhibitory activities against several tumor cell lines indicate that these compounds possess preliminary cytotoxic effects toward cancer cells, their selectivity between tumor and normal cells remains to be further evaluated. Notably, related analogues, including PNSA and PEN-A, have been reported to exhibit cytotoxicity toward the normal human L-02 liver cell line [18,19]. Therefore, the present findings provided an initial evaluation of their bioactive potential, and further investigations will be required to clarify their biological mechanisms and assess their suitability for future therapeutic development.

## 4. Materials and Methods

### 4.1. General Experimental Procedures

Optical rotations were measured with a JASCO P-1020 digital polarimeter (JASCO Corporation, Tokyo, Japan). UV spectra were recorded on a Beckman DU640 spectrophotometer (Beckman Coulter, CA, USA), whereas IR spectra were obtained with a Nicolet NEXUS 470 spectrophotometer (Thermo Nicolet Corporation, Madison, WI, USA) in KBr disks. HPLC-MS analysis was performed using an UHPLC system (Ultimate 3000, Thermo Scientific, Bremen, Germany) with a C18 column (ACQUITY UPLC BEH 1.7 μm, 2.1 × 100 mm, Waters, MA, USA) coupled to a hybrid quadrupole-Orbitrap mass spectrometer (Thermo Fisher Scientific, Bremen, Germany). Injection volume: 2 μL; column temperature: 35 °C; mobile phase composition (positive ion mode): A: methanol, B: 0.1% formic acid solution. Column chromatography (CC) was performed on silica gel (100–400 mesh, Qingdao Marine Chemical Factory, Qingdao, China), Sephadex LH-20 (Amersham Biosciences), and ODS resin (50 mm, Merck, Darmstadt, Germany). Preparative HPLC was performed using a C18 column (YMC-Pack ODS-A, 10 × 250 mm, 5 μm, 3 mL/min, Kyoto, Japan). ^1^H NMR, ^13^C NMR, DEPT, and 2D NMR spectra were recorded on an Agilent 500 MHz DD2 spectrometer (Agilent Technologies, CA, USA); ESIMS was measured on a Micromass Q-TOF Ultima Global GAA076 LC mass spectrometer (Waters, MA, USA); HRESIMS was obtained with a Micromass EI-4000 (Waters, MA, USA); and CD spectra were measured on a JASCO J-715 (JASCO Corporation, Tokyo, Japan) or Chirascan CD spectropolarimeter (Applied Photophysics Ltd., Surrey, UK).

### 4.2. Strains and Culture Conditions

The fungal strain HDN13-309 (GenBank accession number KM659023) was isolated from the root of the mangrove plant *Sonneratia caseolaris* after disinfection and was identified as *Penicillium janthinellum* by ITS sequence. A voucher specimen was deposited in our laboratory at −20 °C, and the working strain was prepared on potato dextrose agar slants and stored at 4 °C. The fungus was cultured for two months under static conditions at 28 °C in 1000 mL Erlenmeyer flasks containing 300 mL of liquid medium containing maltose (2%), mannitol (2%), glucose (1%), sodium glutamate (1%), yeast extract (0.3%), corn syrup (0.1%), KH_2_PO_4_ (0.05%), and MgSO_4_∙7H_2_O (0.03%) dissolved in naturally collected seawater (Huiquan Bay, Yellow Sea, Qingdao, China).

### 4.3. Isolation and Purification

The crude extract was subjected to column chromatography on silica gel (300–400 mesh) and separated by gradient elution of MeOH and CH_2_Cl_2_, labeled as Fractions 1 to 7. Combined with LC-MS analysis, Fractions 2, 5, and 6 were further purified using a C18 column. Fraction 6 yielded four subfractions using a stepwise gradient of 20–80% MeOH. Fr.6-4 was then subjected to semipreparative HPLC (45:55 MeOH-H_2_O, 3 mL/min), resulting in the isolation of compound **1** (3 mg). Fraction 2 was separated into five parts by chromatography on a C18 column using stepwise gradient elution of 20–80% MeOH-H_2_O. Fr.2-2 was applied to semipreparative HPLC (30:70 MeCN-H_2_O, 3 mL/min) to afford compounds **2** (5 mg) and **3** (3 mg). Fraction 5 was separated into six parts by chromatography on a C18 column using stepwise gradient elution of 20–80% MeOH-H_2_O. Fr.5-6 was separated by semipreparative HPLC (30:70 MeCN-H_2_O, 3 mL/min), resulting in the isolation of compound **4** (6 mg). Similarly, Fr.5-4 was purified via semipreparative HPLC with a MeCN-H_2_O (35:65) solvent system at the same flow rate, yielding compound **5** (6 mg).

Penicisulfuranol G (**1**): pale yellow powder; [*α*]^21^_D_ −30.9 (*c* 0.1, MeOH); UV (MeOH) *λ*_max_ (log *ε*) 202 (3.31) nm; ECD (0.91 × 10^−3^ M, MeOH) *λ*_max_ (Δ*ε*) 308 (−3.67), 283 (−2.33), 214 (−17.65) nm; IR (KBr) *ʋ*_max_ 3503, 1658, 1623, 1601, 1438, 1384, 1093, 1035, 709, 671, 652 cm^−1^; ^1^H and ^13^C NMR data (see Table 2 and Table 3). HRESIMS *m*/*z* 511.0842 [M + H]^+^ (calcd. for C_21_H_22_N_2_O_9_S_2_, 511.0839).

Penicisulfuranol H (**2**): pale yellow powder; [*α*]^21^_D_ −18.3 (*c* 0.1, MeOH); UV (MeOH) *λ*_max_ (log *ε*) 202 (3.31) nm; IR (KBr) *ʋ*_max_ 3503, 1658, 1623, 1601, 1438, 1384, 1093, 1035, 709, 671, 652 cm^−1^; ^1^H and ^13^C NMR data (see Table 1 and Table 2). HRESIMS *m*/*z* 563.1127 [M + Na]^+^ (calcd. for C_23_H_28_N_2_O_9_S_2_Na, 563.1128).

Penicisulfuranol I (**3**): pale yellow powder; [*α*]^21^_D_ −40.8 (*c* 0.1, MeOH); UV (MeOH) *λ*_max_ (log *ε*) 205 (3.32) nm; ECD (0.46 × 10^−3^ M, MeOH) *λ*_max_ (Δ*ε*) 264 (−1.82), 243 (+3.65), 218 (−8.50) nm; IR (KBr) *ʋ*_max_ 3514, 1665, 1628, 1610, 1425, 1383, 1092, 1010, 709, 670, 652 cm^−1^; ^1^H and ^13^C NMR data (see Table 1 and Table 2). HRESIMS *m*/*z* 565.0388 [M + Na]^+^ (calcd. for C_21_H_22_N_2_O_9_S_3_Na, 565.0380).

Penicisulfuranol J (**4**): pale yellow powder; [*α*]^21^_D_ −42.8 (*c* 0.1, MeOH); UV (MeOH) *λ*_max_ (log *ε*) 202 (3.31) nm; ECD (1.07 × 10^−3^ M, MeOH) *λ*_max_ (Δ*ε*) 279 (−0.89), 265 (−0.57), 226 (−7.17); IR (KBr) *ʋ*_max_ 3515, 1663, 1623, 1610, 1424, 1384, 1093, 1010, 709, 670, 652 cm^−1^; ^1^H and ^13^C NMR data (see Table 1 and Table 2). HRESIMS *m*/*z* 489.0394 [M + Na]^+^ (calcd for C_19_H_18_O_8_N_2_NaS_2_, 489.0397).

Penicisulfuranol K (**5**): pale yellow powder; [*α*]^21^_D_ −34.3 (*c* 0.1, MeOH); UV (MeOH) *λ*_max_ (log *ε*) 200 (3.42) nm; ECD (0.97 × 10^−3^ M, MeOH) *λ*_max_ (Δ*ε*) 300 (+0.46), 278 (−0.41), 264 (+0.13), 219 (−8.63), 203 (−3.96) nm; IR (KBr) *ʋ*_max_ 3511, 1661, 1625, 1606, 1446, 1383, 1098, 1030, 709, 670, 652 cm^−1^. ^1^H and ^13^C NMR data (see Table 1 and Table 2). HRESIMS *m*/*z* 535.0272 [M + Na]^+^ (calcd for C_20_H_20_O_8_N_2_NaS_3_, 535.0274).

### 4.4. Computation Section

Conformational searches were performed via molecular mechanics using the MMFF (Merck Molecular Force Field) method in Spartan’14 software (Version 1.1.4) [24]. To obtain the energy-minimized conformers, the resultant corresponding stable conformers (rel. E < 5 kcal mol^−1^) were collected, and the geometries were reoptimized at the B3LYP/6-31+G(d) PCM/MeOH level by using the Gaussian 09 software [25]. Then, the optimized conformers (>5% population) were subjected to the calculations of the ECD spectra using time-dependent DFT (TD-DFT) at B3LYP/6-31G(d) PCM/MeOH (using 30 excited states). The ECD spectra were generated using the program SpecDis (Version 1.53) [26] by applying a Gaussian band shape from dipole-length rotational strengths. The spectra of the conformers were combined using Boltzmann weighting, with the lowest-energy conformations accounting for about 98% of the weights. The calculated spectra were blue-shifted (10–20 nm for compounds **1** and **4**) to facilitate comparison to the experimental data.

### 4.5. Bioassays

The cytotoxic activities of compounds **1**–**5** were evaluated against four human tumor cell lines, including HCT116 (human colorectal cancer cell line), HeLa (human cervical carcinoma cell line), HL-60 (human promyelocytic leukemia cell line), and K562 (human chronic myelogenous leukemia cell line), using the SRB and MTT methods. All four cell lines were purchased from the National Collection of Authenticated Cell Cultures of China. Adriamycin was used as the positive control. For the cytotoxicity assay, suspension cells were seeded in a 96-well plate at a density of 6000 cells per well. Cells were incubated with compounds (dissolved in 1‰ DMSO) for 24 h. Each experiment was repeated three biologically independent times with triplicate technical replicates. The data were expressed as mean ± SD.

## 5. Conclusions

This study on the mangrove endophytic fungus *Penicillium janthinellum* HDN13-309 resulted in the identification and characterization of five novel gliovirin-like alkaloids, penicisulfuranols G–K (**1**–**5**). Penicisulfuranols G and H represented unusual gliovirin analogs characterized by a spirocyclic skeleton and an unprecedented migration of the double bond to the C-5/C-6 positions, while penicisulfuranols G and I–K possessed structurally unusual *α*,*β*-sulfide bridges together with distinctive methoxy substituents. Penicisulfuranols I–K showed noteworthy cytotoxicities against four tumor cell lines (HCT116, HeLa, HL-60, and K562) with IC_50_ values ranging from 0.1 to 9.9 μM. These findings broaden the structural diversity of gliovirin-type epipolythiodioxopiperazines and highlight their potential as promising leads for anticancer drug discovery.

## Figures and Tables

**Table 1:** Cytotoxicity of compounds **1**–**5** against four tumor cell lines.

No.	IC_50_ (μM)			
	HCT116	HeLa	HL-60	K562
Adriamycin	0.20 ± 0.10	0.60 ± 0.11	0.20 ± 0.05	0.30 ± 0.07
**1**	>30.0	>30.0	>30.0	>30.0
**2**	>30.0	>30.0	>30.0	>30.0
**3**	0.10 ± 0.05	0.80 ± 0.13	0.20 ± 0.09	9.90 ± 0.82
**4**	6.90 ± 0.62	3.80 ± 0.40	1.70 ± 0.25	>30.0
**5**	2.70 ± 0.36	6.00 ± 0.49	1.30 ± 0.22	5.70 ± 0.49

**Table 2:** ^13^C NMR (125 MHz) spectroscopic data for compounds **1**–**5** in DMSO-*d*_6_.

No.	1	2	3	4	5
1	165.4, C	163.5, C	163.1, C	165.7, C	163.6, C
2	67.4, C	67.8, C	76.7, C	69.2, C	77.0, C
3	37.6, CH_2_	39.9, CH_2_	37.6, CH_2_	31.2, CH_2_	36.6, CH_2_
4	68.6, C	68.3, C	71.2, C	71.1, C	72.1, C
5	134.7, CH	126.6, CH	66.8, CH	74.9, CH	73.9, CH
6	127.6, CH	134.5, CH	126.4, CH	130.0, CH	129.9, CH
7	66.6, CH	65.9, CH	131.7, CH	129.1, CH	129.1, CH
8	66.3, CH	65.8, CH	64.8, CH	65.0, CH	65.5, CH
9	82.6, CH	85.3, CH	87.4, CH	86.5, CH	87.3, CH
10		15.7, CH_3_			
1′	162.6, C	158.6, C	161.2, C	162.2, C	161.6, C
2′	99.7, C	100.8, C	101.3, C	98.8, C	101.0, C
3′	51.3, CH	55.4, CH	65.1, CH	51.9, CH	65.2, CH
4′	116.0, C	120.2, C	118.7, C	123.5, C	126.0, C
5′	119.4, CH	119.4, CH	118.3, CH	115.1, CH	115.9, CH
6′	107.2, CH	107.0, CH	106.5, CH	123.5, CH	123.3, CH
7′	154.6, C	153.6, C	154.3, C	118.7, CH	114.2, CH
8′	133.4, C	132.7, C	133.3, C	142.1, C	144.0, C
9′	150.2, C	150.4, C	150.6, C	145.6, C	146.5, C
10′	28.7, CH_3_	28.8, CH_3_	28.7, CH_3_	28.8, CH_3_	28.7, CH_3_
11′	56.7, CH_3_	56.7, CH_3_	56.7, CH_3_		56.2, CH_3_
12′	60.8, CH_3_	60.7, CH_3_	60.9, CH_3_		
13′		14.4, CH_3_			

**Table 3:** ^1^H NMR (500 MHz) spectroscopic data for compounds **1**−**5** in DMSO-*d*_6_.

No.	1	2	3	4	5
	(*J* in Hz)				
1					
2					
3	a 2.20, d (15.7) b 2.31, d (15.7)	a 2.14, d (15.0) b 2.34, d (15.0)	a 2.53, d (14.9) b 2.31, d (14.9)	a 2.11, d (16.4) b 2.01, d (16.4)	a 2.50, d (15.4) b 2.06, d (15.4)
4					
5	5.56, d (10.0)	5.54, d (9.9)	4.86, d (2.3)	4.17, br s	4.19, d (2.1)
6	5.60, dd (10.0, 4.4)	5.61, dd (10.0, 4.9)	5.55, d (10.2)	5.42, d (10.4)	5.41, d (10.5)
7	4.00, t (4.4)	4.14, t (4.9)	5.59, d (10.2)	5.49, d (10.4)	5.46, d (10.5)
8	3.76, dd (4.4, 9.9)	3.74, dd (4.9, 10.8)	4.45, brs	4.28, m	4.29, brs
9	4.12, d (9.9)	4.17, d (10.8)	4.11, d (7.2)	3.97, d (7.3)	4.02, d (7.2)
10		2.22, s			
1′					
2′					
3′	5.22, s	4.97, s	5.90, s	5.26, s	6.00, s
4′					
5′	6.85, d (8.4)	6.94, d (8.4)	6.74, d (8.3)	6.62, d (6.5)	7.01, d (8.4)
6′	6.66, d (8.4)	6.69, d (8.4)	6.61, d (8.3)	6.81, m	6.93, d (8.4)
7′				6.83, m	6.68, d (8.4)
8′					
9′					
10′	2.96, s	2.83, s	3.05, s	2.97,s	3.04, s
11′	3.77, s	3.78, s	3.77, s		
12′	3.80, s	3.69, s	3.72, s		3.79, s
13′		2.06, s			
4-OH	5.33, br s	5.46, s	6.18,s	5.08, br s	5.69, s
5-OH			4.06, d (5.2)	5.28, br s	5.31, d (4.2)
7-OH		4.51, d (2.3)			
8-OH		4.95, d (4.7)	5.41, d (6.7)	5.35, br s	5.26, d (6.6)
8′-OH				9.89, br s	

## Data Availability

The original contributions presented in this study are included in the article/Supplementary Material. Further inquiries can be directed to the corresponding author(s).

## Supplementary Materials

The following supporting information can be downloaded at https://www.mdpi.com/article/10.3390/md24080258/s1. Figure S1: HPLC-MS analysis of crude extract of the strain; Figures S2 and S3: Conformations of lowest-energy conformers of compounds **1** and **4**; Figure S4: Structure of penicisulfuranol B and experimental ECD spectra of penicisulfuranol B and compound **3**; Figures S5–S37: The 1D, 2D NMR, and HRESIMS spectra of compounds **1**–**5**; Table S1 and S2: Cartesian coordinates of the low-energy reoptimized conformers of compounds **1** and **4**.
