# Supplementary material for: Gliovirin-like Alkaloids with Spirocyclic Skeletons from the Mangrove Endophytic Fungus Penicillium janthinellum HDN13-309
Source: Mar Drugs. 2026 Jul 26;24(8):258. doi: 10.3390/md24080258 (PMC13514329; doi:10.3390/md24080258)
Supplement: Supplementary file 1 [file marinedrugs-24-00258-s001.zip › marinedrugs-4409087-supplementary.pdf]

Supporting Information for

Gliovirin-like Alkaloids with Spirocyclic Skeletons from the Mangrove Endophytic Fungus *Penicillium janthinellum* HDN13-309

Haotian Wang [1,†], Runyu Wu [1,†], Lanying Li [1], Huimin Feng[2], Yixuan Zhang [1], Yajuan Cong [1], Dehai Li [2,*], Hao Liu [1,*] and Meilin Zhu [1,*]

[1] School of Pharmacy, Bengbu Medical University, Bengbu 233030, China; haotian@bbmu.edu.cn (H.W.)
[2] School of Medicine and Pharmacy, Ocean University of China, Qingdao 266003, China
* Correspondence: dehaili@ouc.edu.cn (D.L.); liuhao6886@bbmu.edu.cn (H.L.); meilin0402@bbmu.edu.cn (M.Z.)
† These authors contributed equally to this work.

Contents

Figure S1. HPLC-MS analysis of crude extract of the strain (Positive ion) 1
Figure S2. Conformations of lowest-energy conformers of **1**......................................1
Figure S3. Conformations of lowest-energy conformers of **4**......................................2
Figure S4. Experimental ECD spectra of penicisulfuranol B and **3**.............................2
Figure S5. ^{1}H NMR (500 MHz) spectrum of penicisulfuranol G (**1**) in d_6-DMSO......3
Figure S6. ^{13}C NMR (125 MHz) spectrum of penicisulfuranol G (**1**) in d_6-DMSO.....3
Figure S7. HSQC spectrum of penicisulfuranol G (**1**) in d_6-DMSO............................4
Figure S8. HMBC spectrum of penicisulfuranol G (**1**) in d_6-DMSO...........................4
Figure S9. NOESY spectrum of penicisulfuranol G (**1**) in d_6-DMSO..........................5
Figure S10. HRESIMS spectrum of penicisulfuranol G (**1**) 5
Figure S11. ^{1}H NMR (500 MHz) spectrum of penicisulfuranol H (**2**) in d_6-DMSO....6
Figure S12. ^{13}C NMR (125 MHz) spectrum of penicisulfuranol H (**2**) in d_6-DMSO...6
Figure S13. HSQC spectrum of penicisulfuranol H (**2**) in d_6-DMSO..........................7
Figure S14. HMBC spectrum of penicisulfuranol H (**2**) in d_6-DMSO.........................7
Figure S15. NOE spectrum of penicisulfuranol H (**2**) in d_6-DMSO............................8
Figure S16. HRESIMS spectrum of penicisulfuranol H (**2**) 8
Figure S17. ^{1}H NMR (500 MHz) spectrum of penicisulfuranol I (**3**) in d_6-DMSO......9
Figure S18. ^{13}C NMR (125 MHz) spectrum of penicisulfuranol I (**3**) in d_6-DMSO.....9
Figure S19. ^{1}H-^{1}H COSY spectrum of penicisulfuranol I (**3**) in d_6-DMSO...............10
Figure S20. HSQC spectrum of penicisulfuranol I (**3**) in d_6-DMSO..........................10
Figure S21. HMBC spectrum of penicisulfuranol I (**3**) in d_6-DMSO.........................11
Figure S22. NOESY spectrum of penicisulfuranol I (**3**) in d_6-DMSO.......................11
Figure S23. HRESIMS spectrum of penicisulfuranol I (**3**) 12
Figure S24. ^{1}H NMR (500 MHz) spectrum of penicisulfuranol J (**4**) in d_6-DMSO....12
Figure S25. ^{13}C NMR (125 MHz) spectrum of penicisulfuranol J (**4**) in d_6-DMSO...13
Figure S26. ^{1}H-^{1}H COSY spectrum of penicisulfuranol J (**4**) in d_6-DMSO...............13
Figure S27. HSQC spectrum of penicisulfuranol J (**4**) in d_6-DMSO..........................14
Figure S28. HMBC spectrum of penicisulfuranol J (**4**) in d_6-DMSO.........................14
Figure S29. NOESY spectrum of penicisulfuranol J (**4**) in d_6-DMSO.......................15
Figure S30. HRESIMS spectrum of penicisulfuranol J (**4**) 15

Figure S31. ^{1}H NMR (500 MHz) spectrum of penicisulfuranol K (**5**) in d_6-DMSO..16

Figure S32. ^{13}C NMR (125 MHz) spectrum of penicisulfuranol K (**5**) in d_6-DMSO.16

Figure S33. ^{1}H-^{1}H COSY spectrum of penicisulfuranol K (**5**) in d_6-DMSO.............17

Figure S34. HSQC spectrum of penicisulfuranol K (**5**) in d_6-DMSO.........................17

Figure S35. HMBC spectrum of penicisulfuranol K (**5**) in d_6-DMSO........................18

Figure S36. NOESY spectrum of penicisulfuranol K (**5**) in d_6-DMSO.......................18

Figure S37. HRESIMS spectrum of penicisulfuranol K (**5**).......................................19

Table S1. Cartesian coordinates of the low-energy reoptimized conformers of **1**........19

Table S2. Cartesian coordinates of the low-energy reoptimized conformers of **4**........26

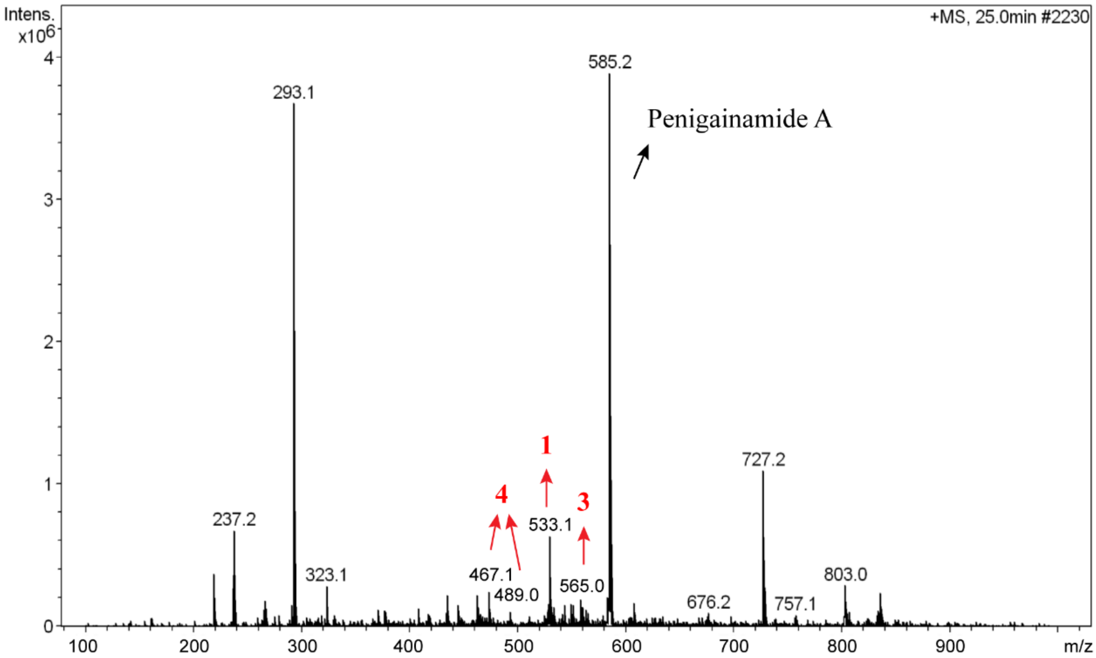

Figure S1. HPLC-MS analysis of crude extract of the strain (Positive ion).

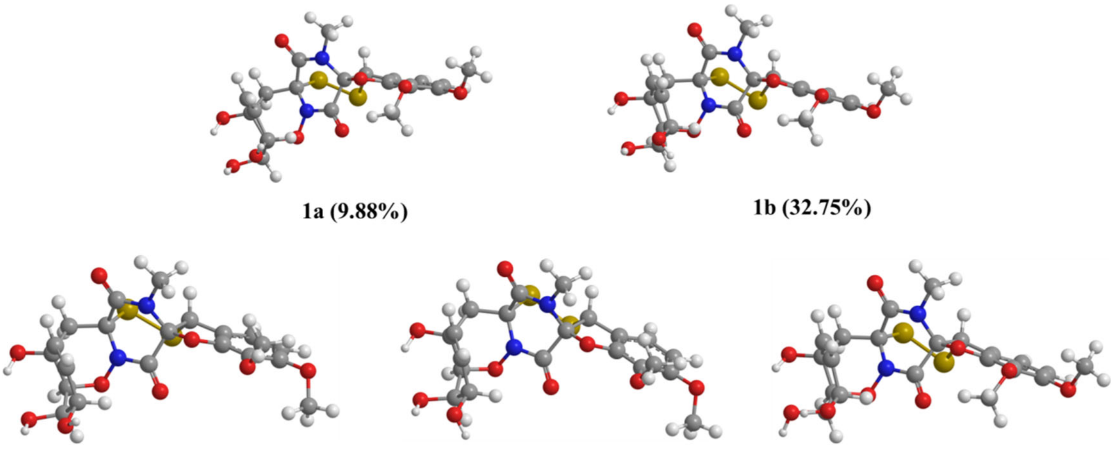

1a (9.88%) **1b** (32.75%)

1c (8.15%) **1d** (26.96%) **1e** (22.27%)

Figure S2. Conformations of lowest-energy conformers (>5% population) of compound **1**.

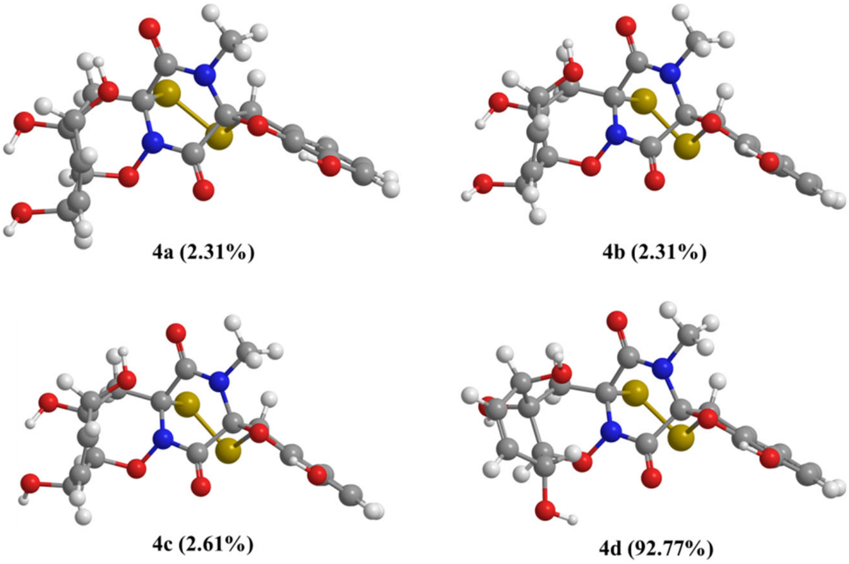

Figure S3. Conformations of lowest-energy conformers of compound **4**.

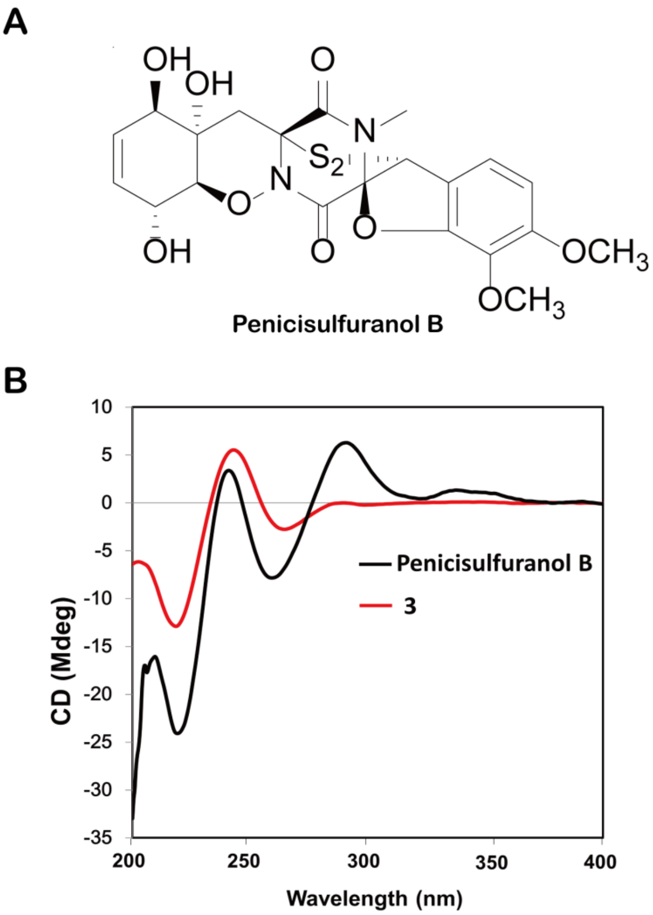

Figure S4. Structure of penicisulfuranol B (A) and experimental ECD spectra of penicisulfuranol B and compound **3** (B).

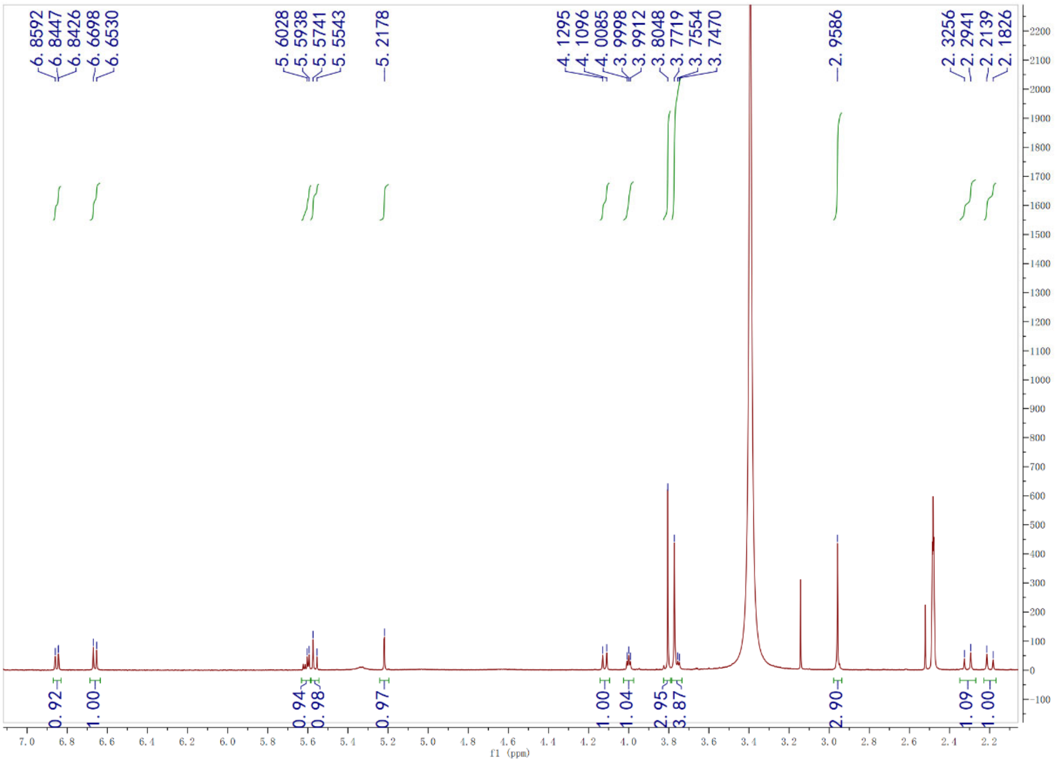

Figure S5. ^{1}H NMR (500 MHz) spectrum of penicisulfuranol G (**1**) in *d*$_6$-DMSO

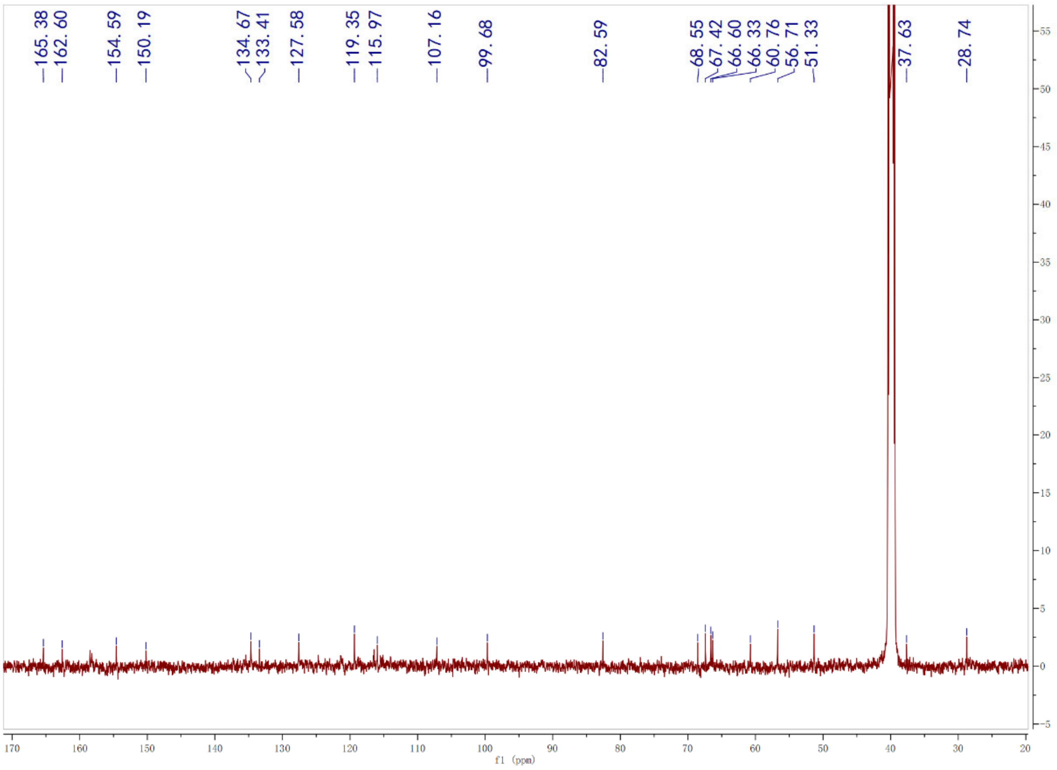

Figure S6. ^{13}C NMR (125 MHz) spectrum of penicisulfuranol G (**1**) in *d*$_6$-DMSO

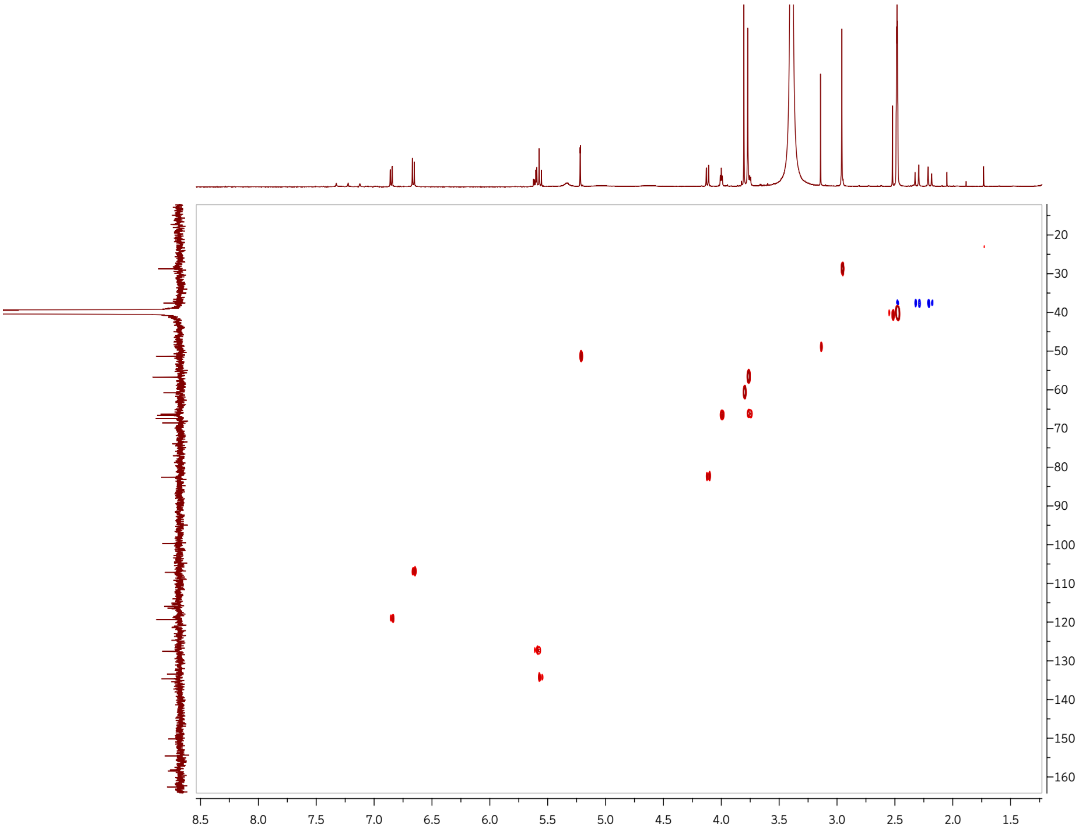

Figure S7. HSQC spectrum of penicisulfuranol G (**1**) in d_6-DMSO

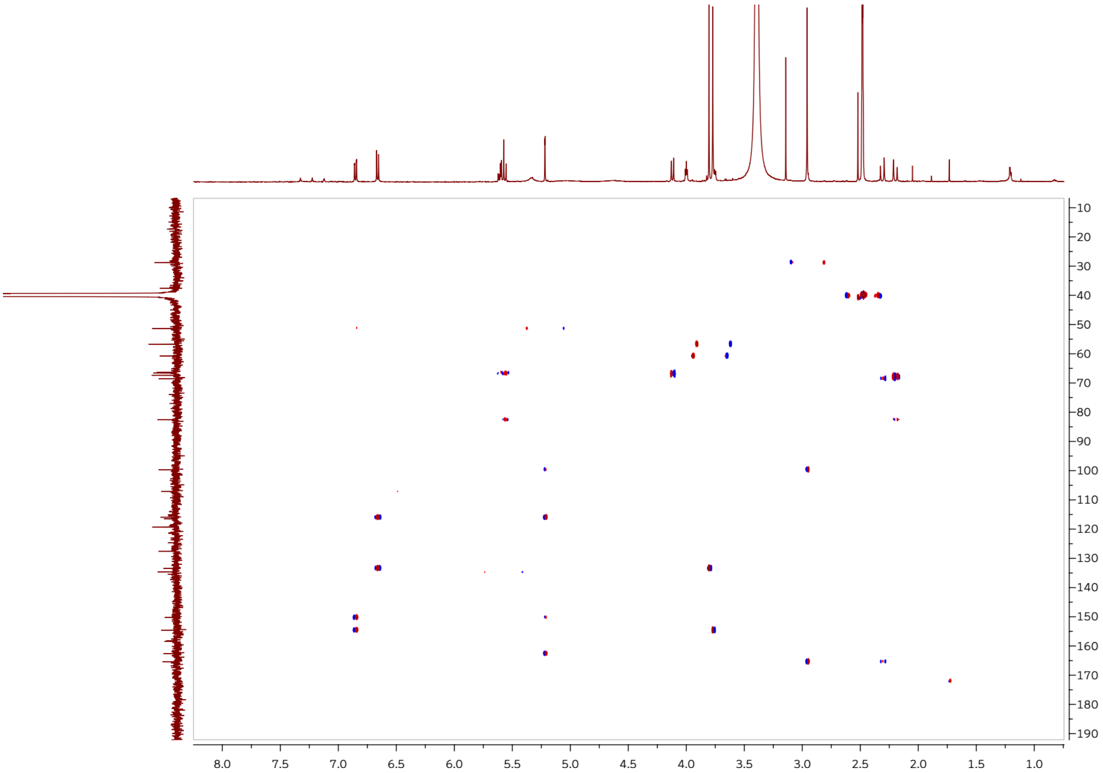

Figure S8. HMBC spectrum of penicisulfuranol G (**1**) in d_6-DMSO

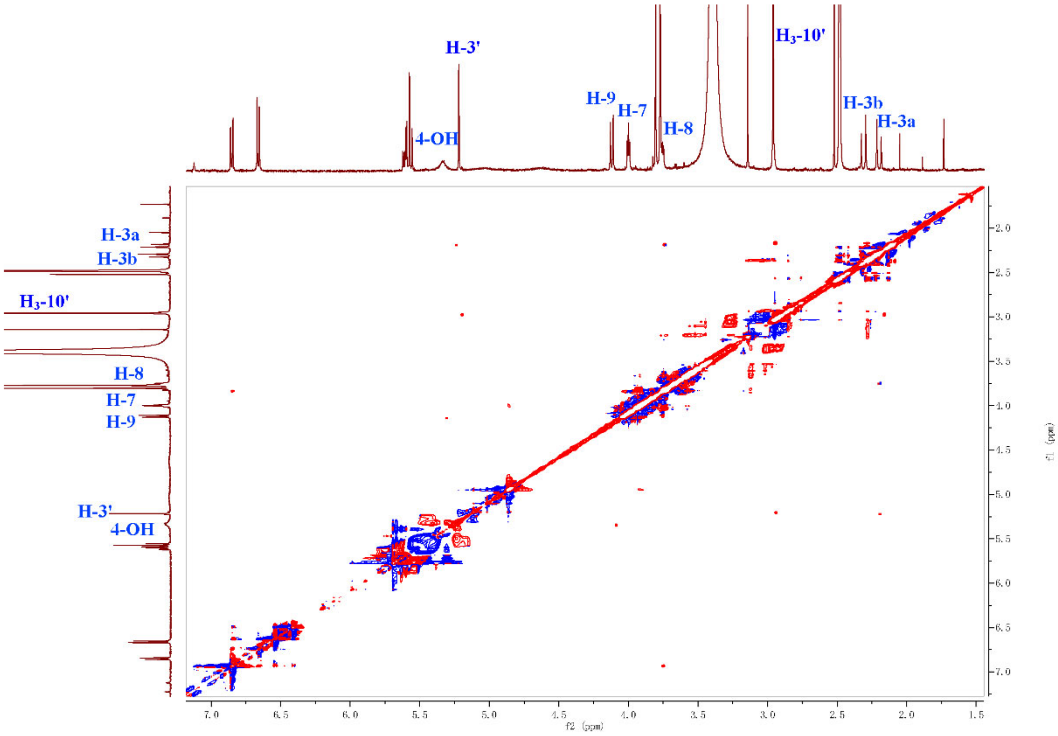

Figure S9. NOESY spectrum of penicisulfuranol G (**1**) in d_6-DMSO

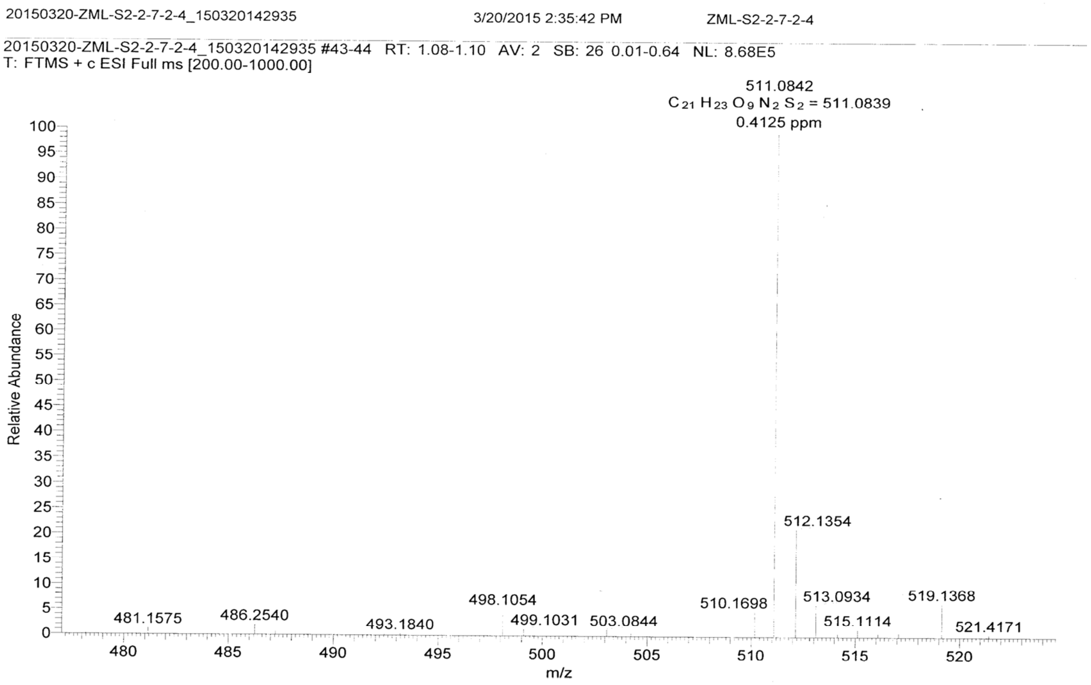

Figure S10. HRESIMS spectrum of penicisulfuranol G (**1**)

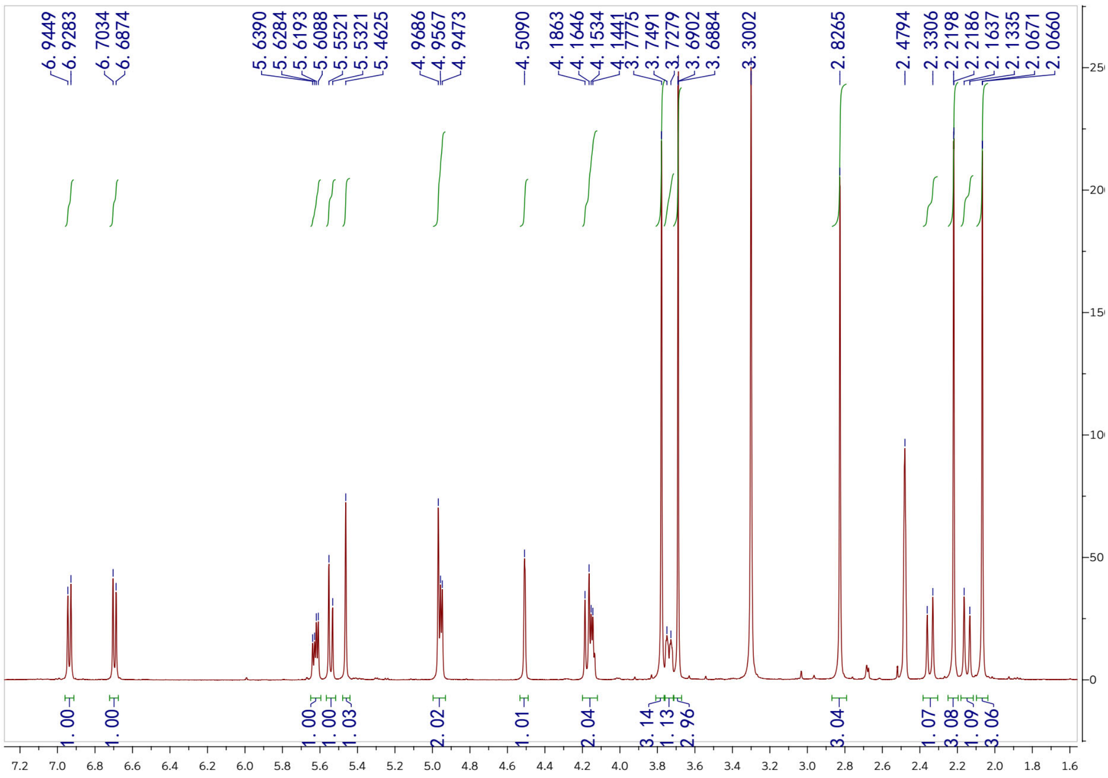

Figure S11. ^{1}H NMR (500 MHz) spectrum of penicisulfuranol H (**2**) in d_6-DMSO

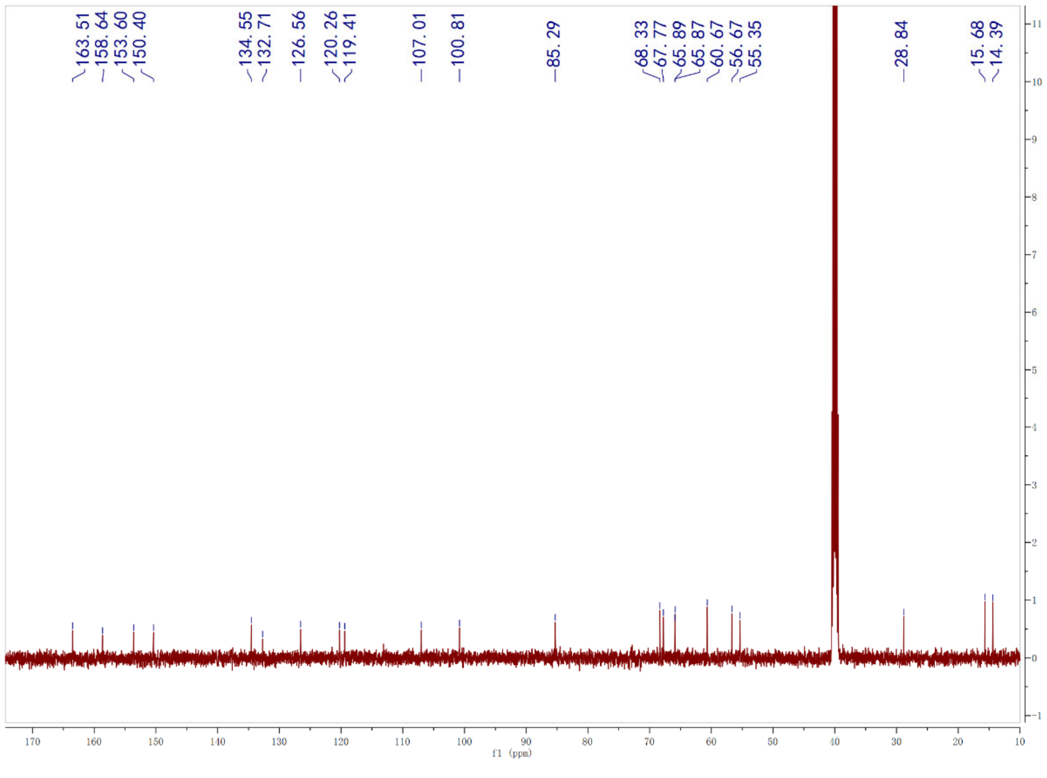

Figure S12. ^{13}C NMR (125 MHz) spectrum of penicisulfuranol H (**2**) in d_6-DMSO

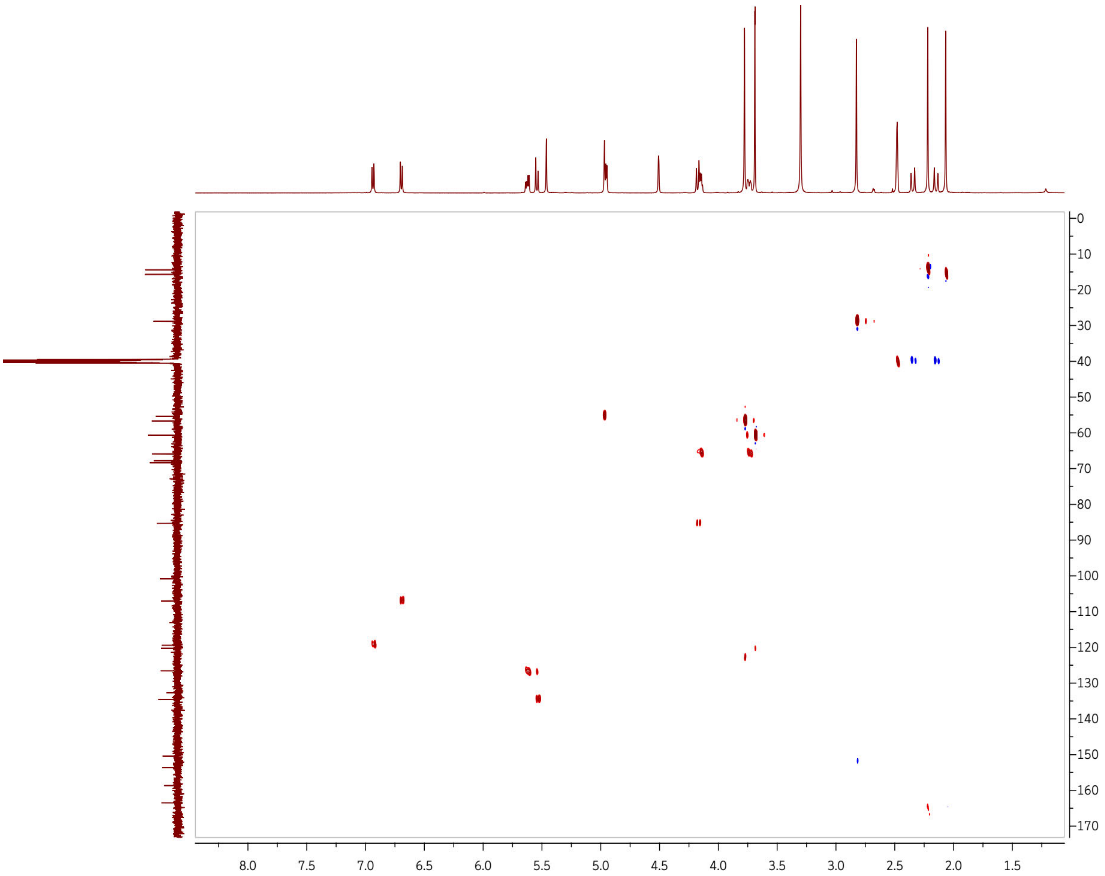

Figure S13. HSQC spectrum of penicisulfuranol H (**2**) in d_6-DMSO

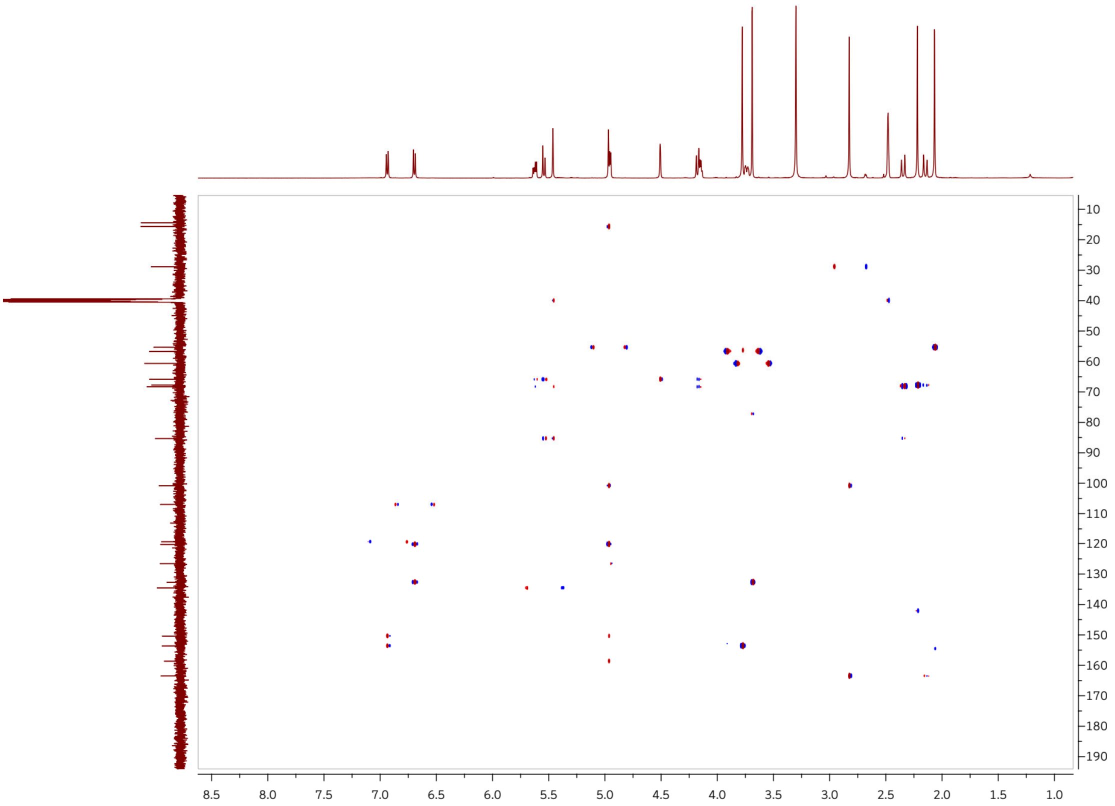

Figure S14. HMBC spectrum of penicisulfuranol H (**2**) in d_6-DMSO

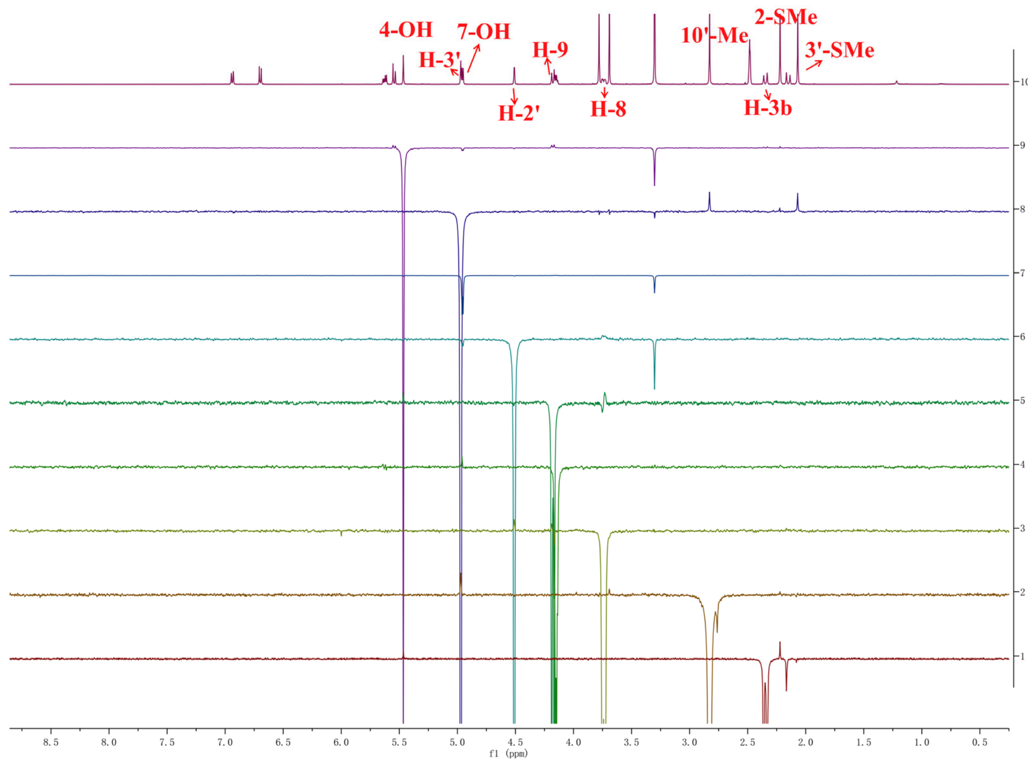

Figure S15. NOE spectrum of penicisulfuranol H (**2**) in d_6-DMSO

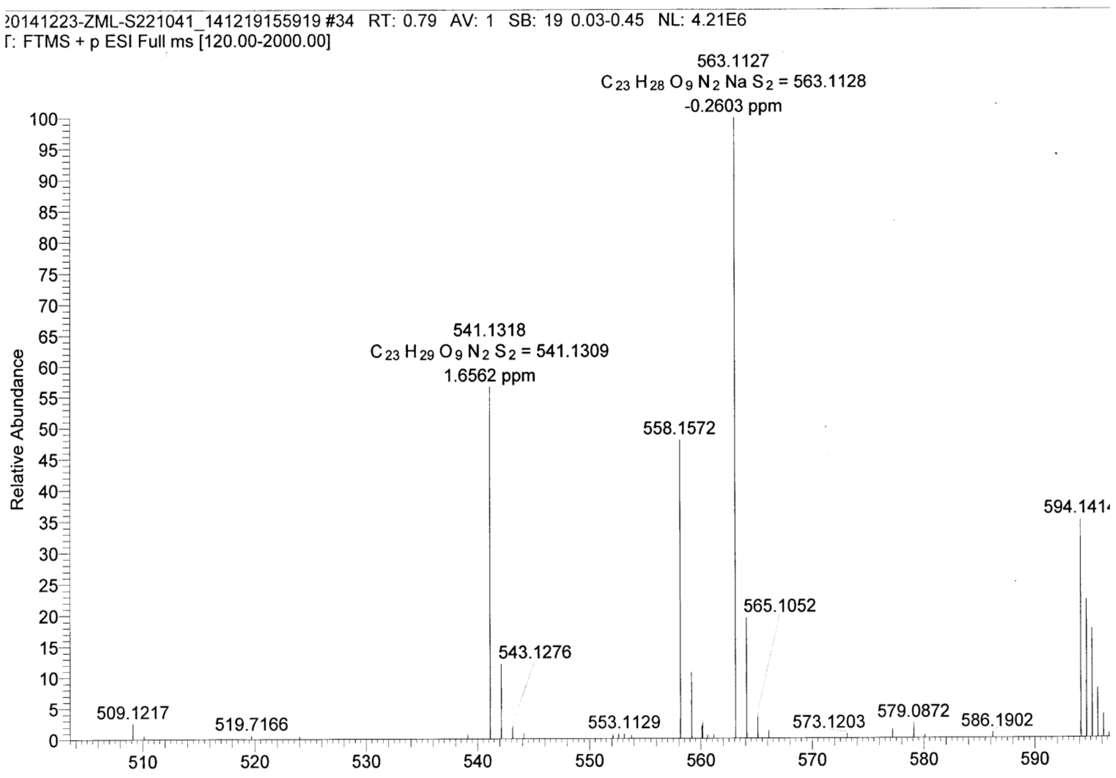

Figure S16. HRESIMS spectrum of penicisulfuranol H (**2**)

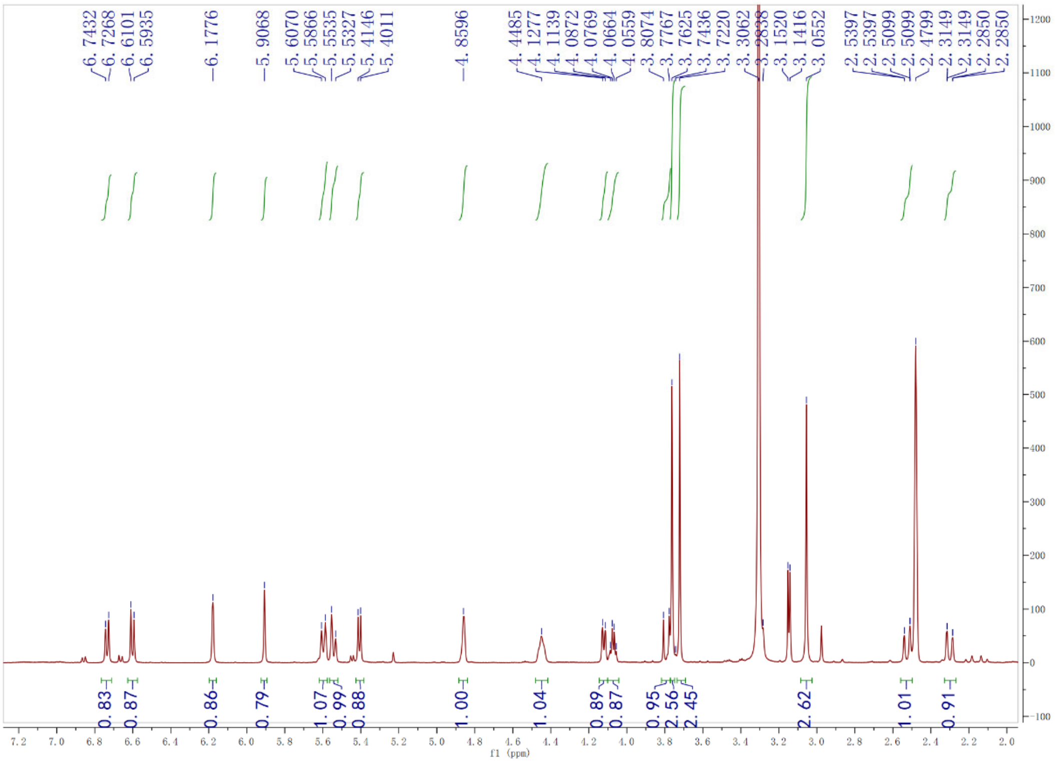

Figure S17. ^{1}H NMR (500 MHz) spectrum of penicisulfuranol I (**3**) in d_6-DMSO

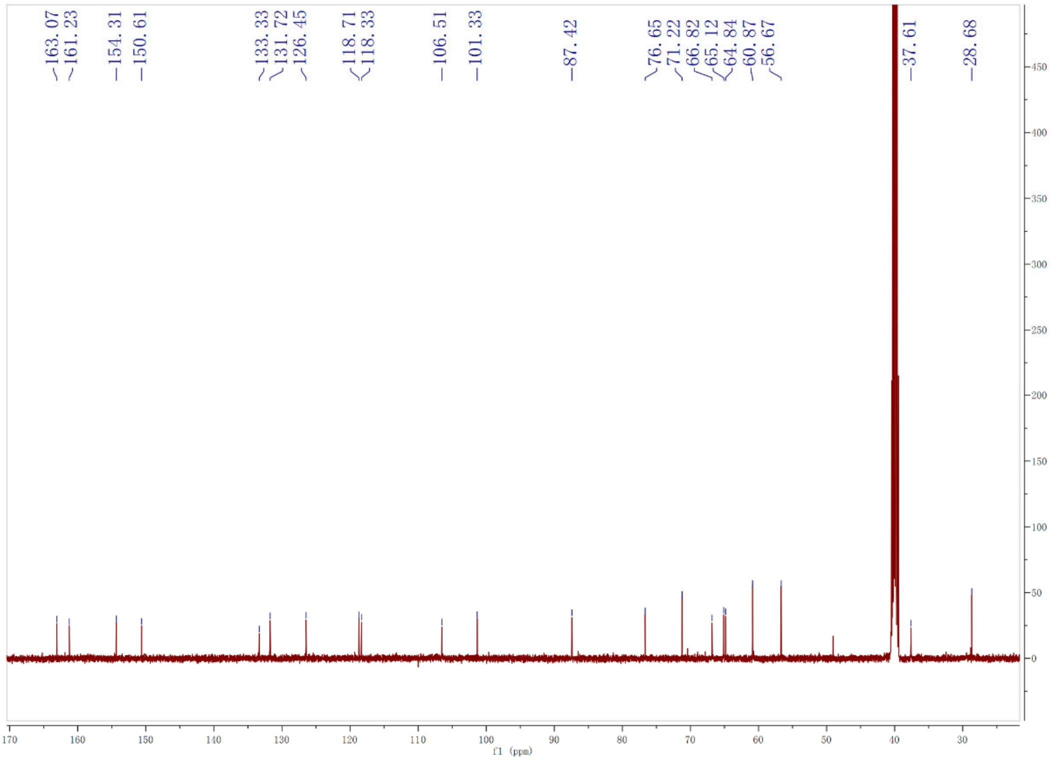

Figure S18. ^{13}C NMR (125 MHz) spectrum of penicisulfuranol I (**3**) in d_6-DMSO

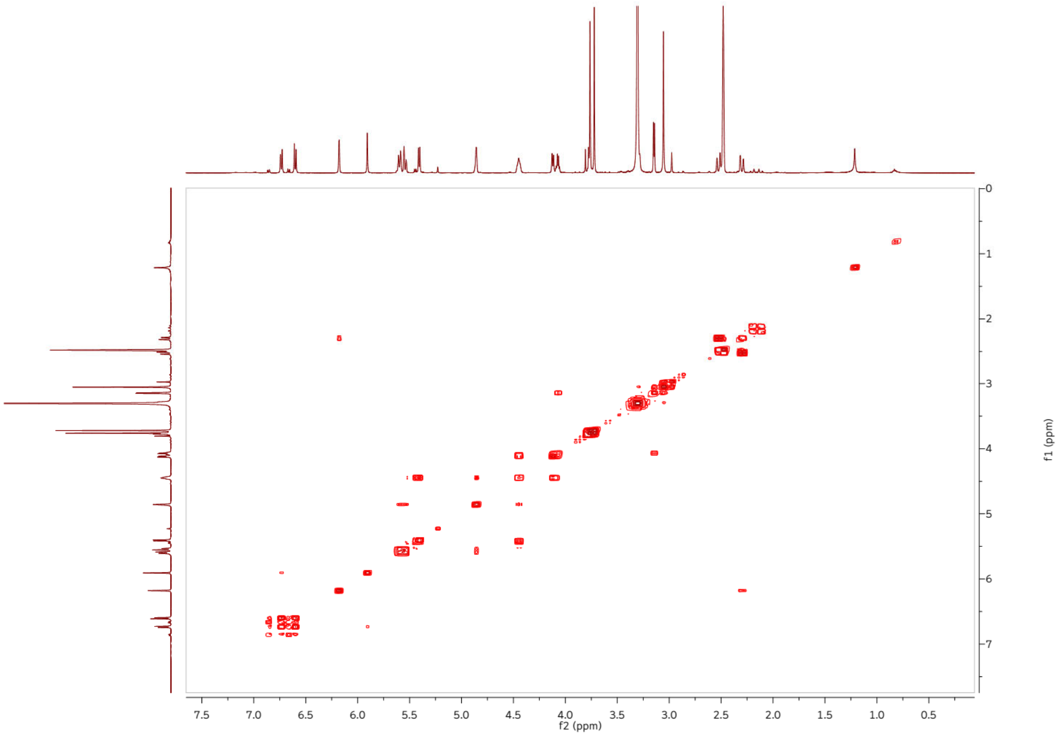

Figure S19. ^{1}H-^{1}H COSY spectrum of penicisulfuranol I (**3**) in d_6-DMSO

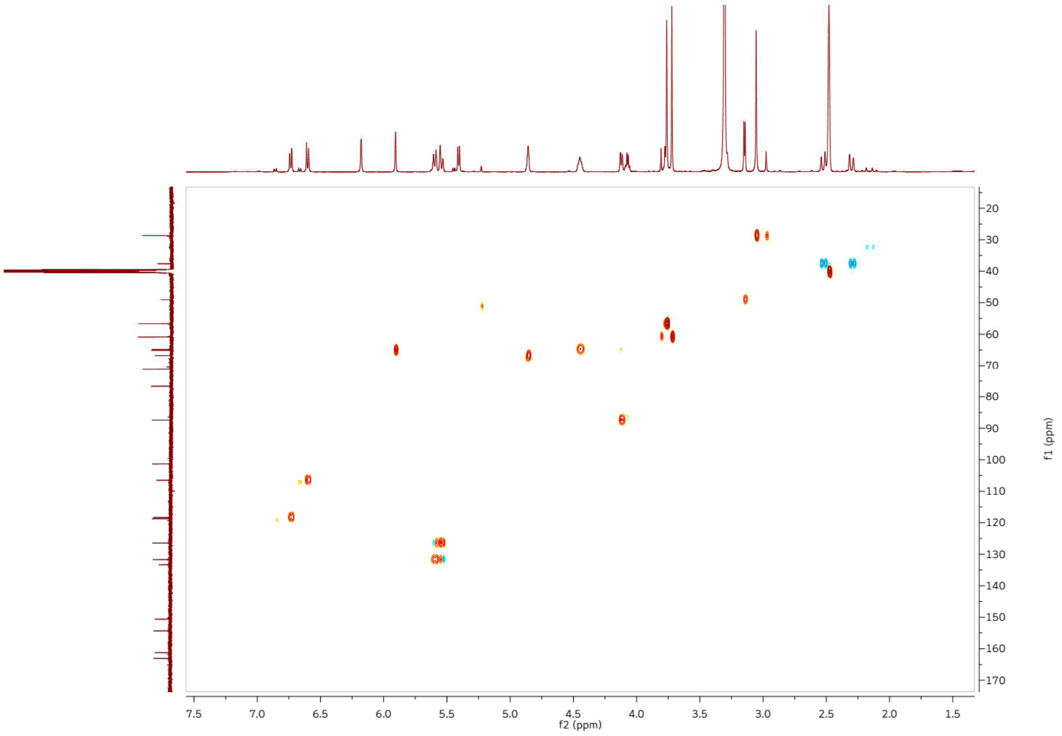

Figure S20. HSQC spectrum of penicisulfuranol I (**3**) in d_6-DMSO

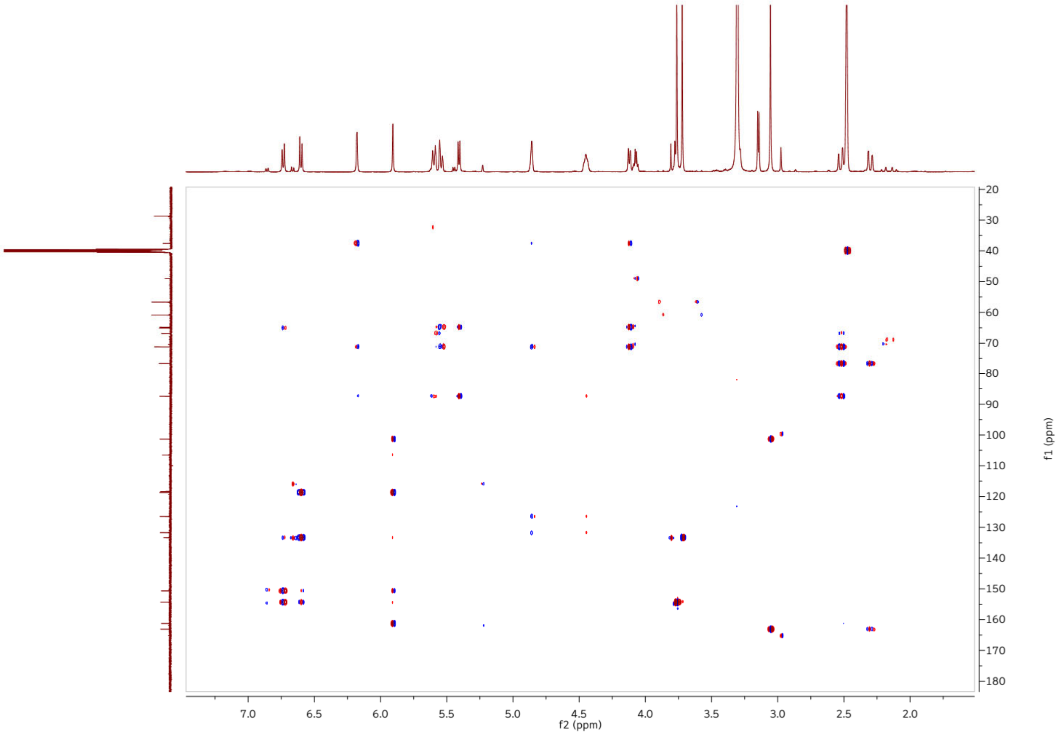

Figure S21. HMBC spectrum of penicisulfuranol I (**3**) in *d*6-DMSO

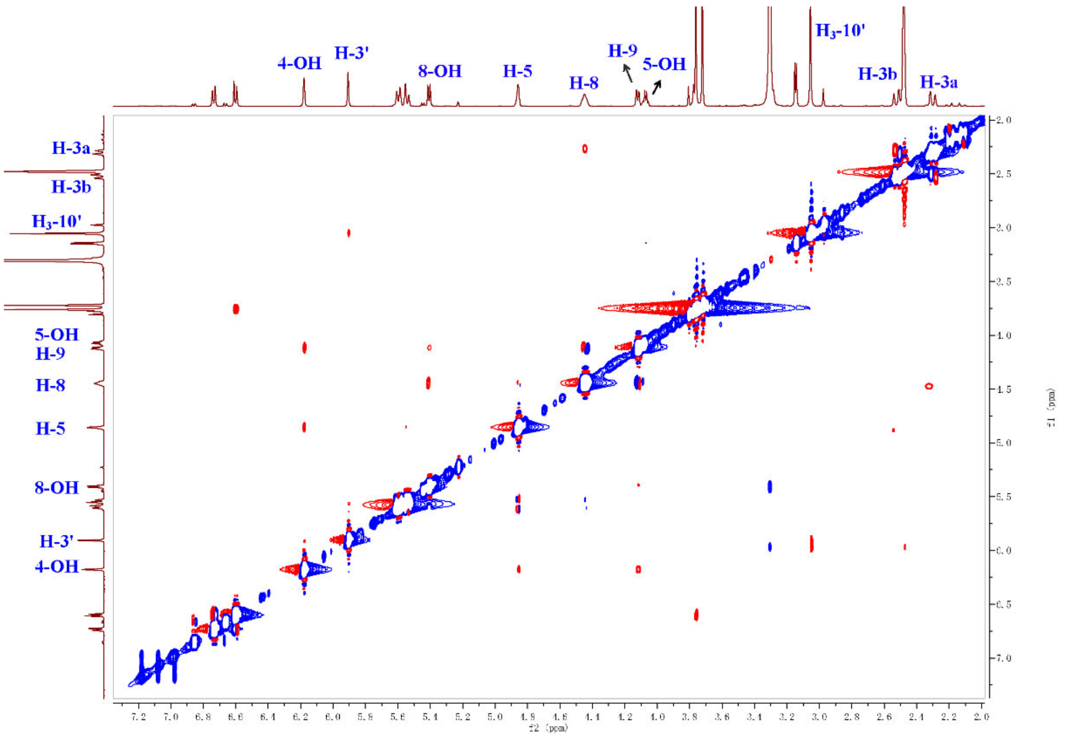

Figure S22. NOESY spectrum of penicisulfuranol I (**3**) in *d*6-DMSO

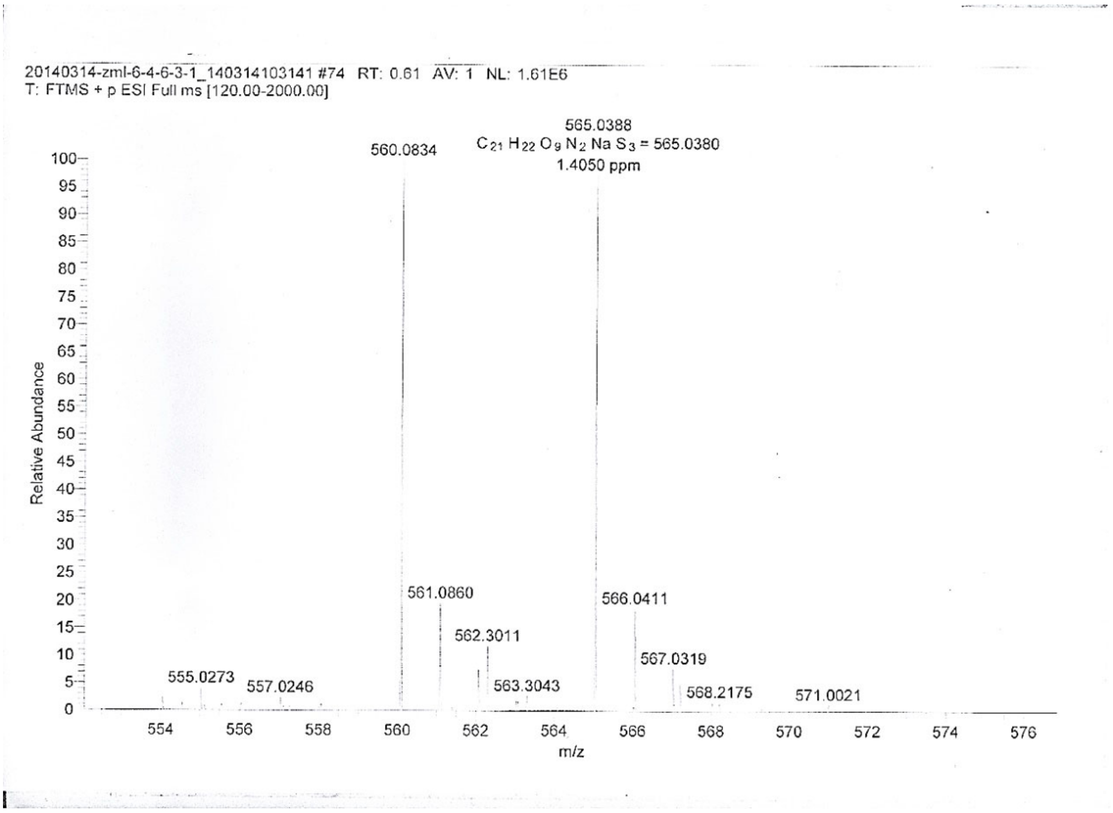

Figure S23. HRESIMS spectrum of penicisulfuranol I (**3**)

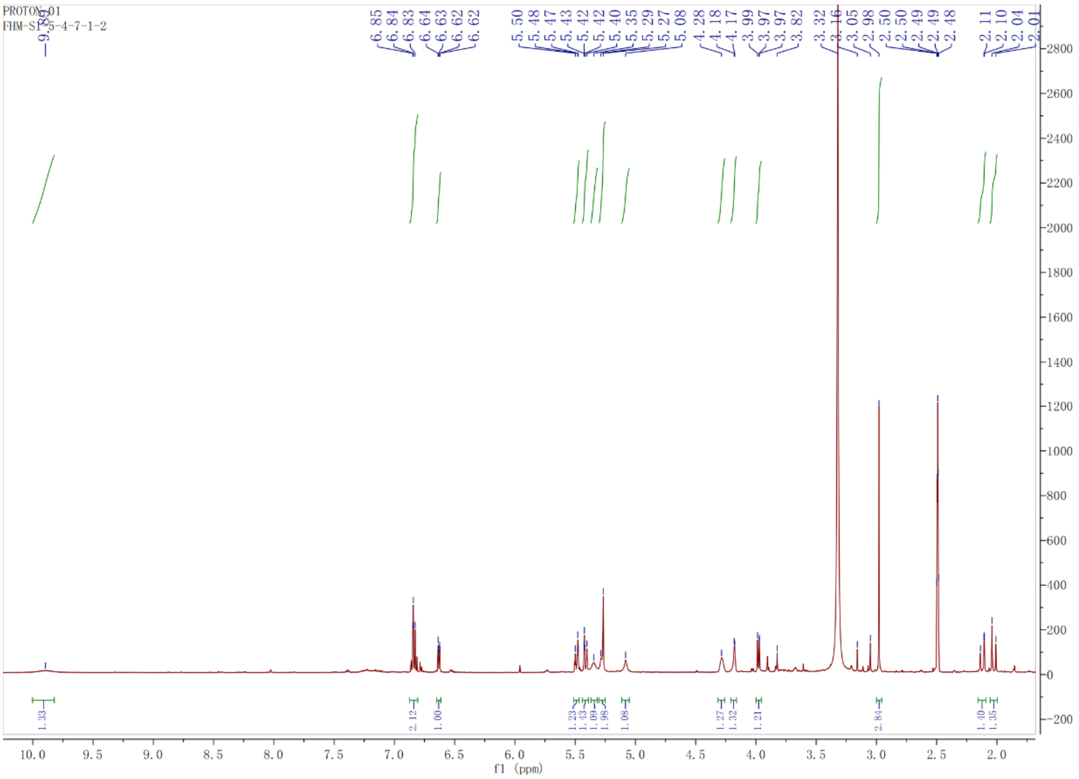

Figure S24. ^{1}H NMR (500 MHz) spectrum of penicisulfuranol J (**4**) in d_6-DMSO

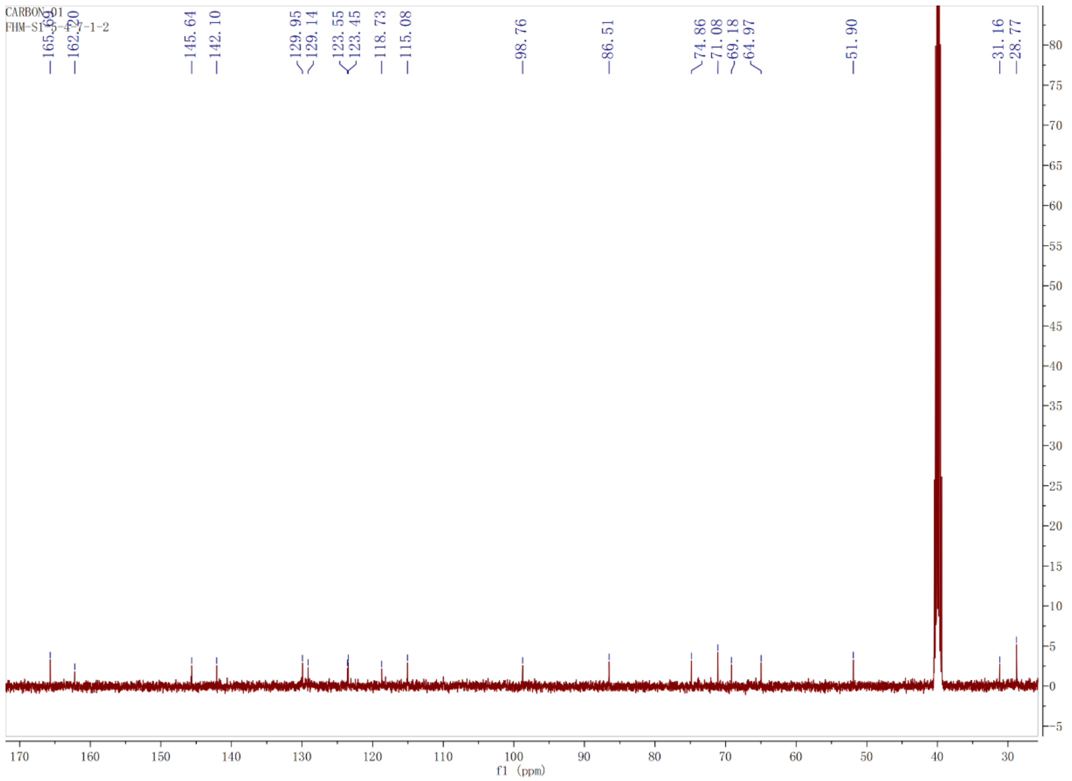

Figure S25. ^{13}C NMR (125 MHz) spectrum of penicisulfuranol J (**4**) in d_6-DMSO

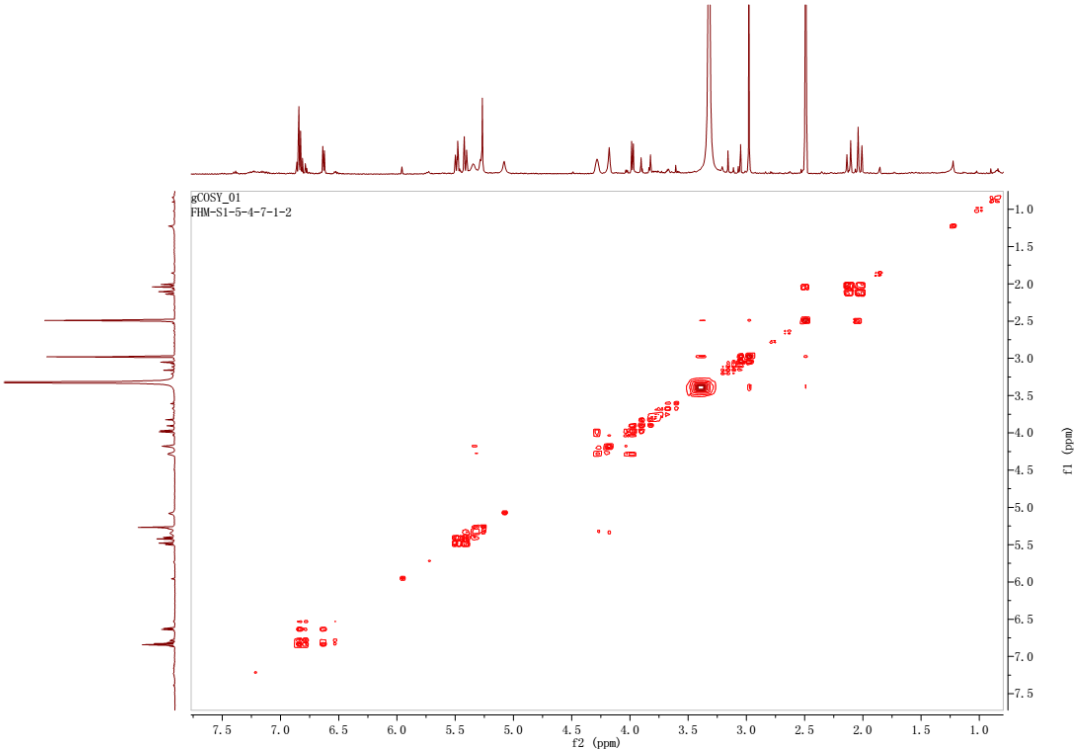

Figure S26. ^{1}H-^{1}H COSY spectrum of penicisulfuranol J (**4**) in d_6-DMSO

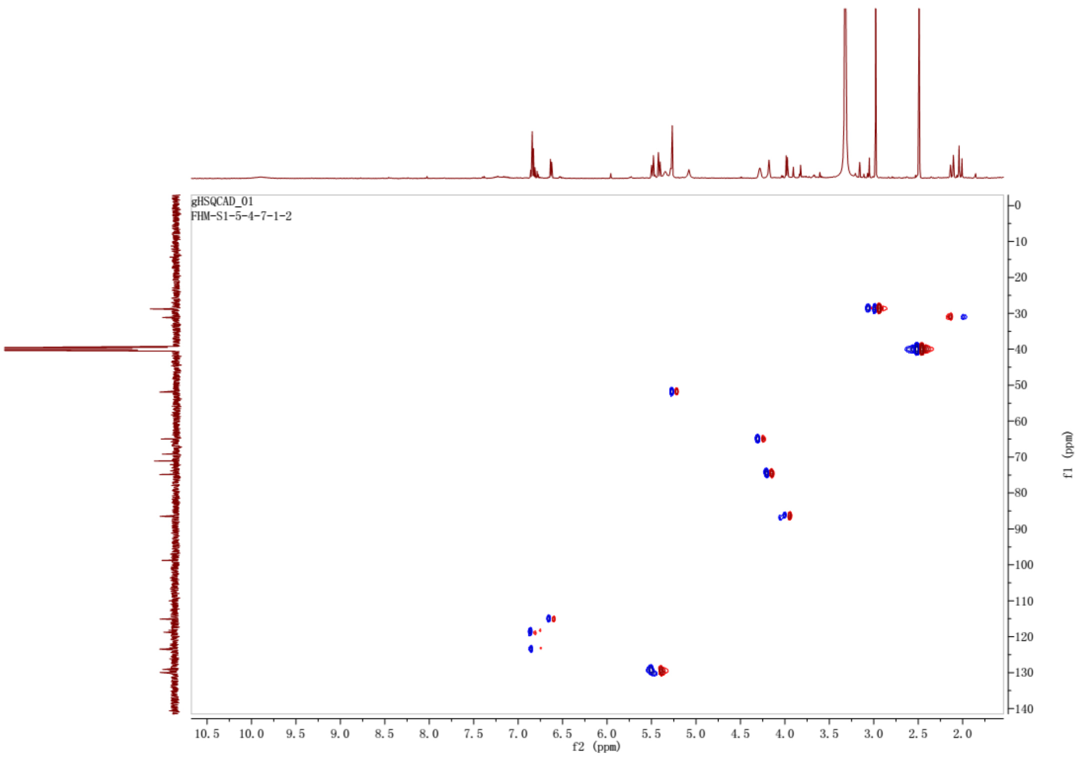

Figure S27. HSQC spectrum of penicisulfuranol J (**4**) in *d*6-DMSO

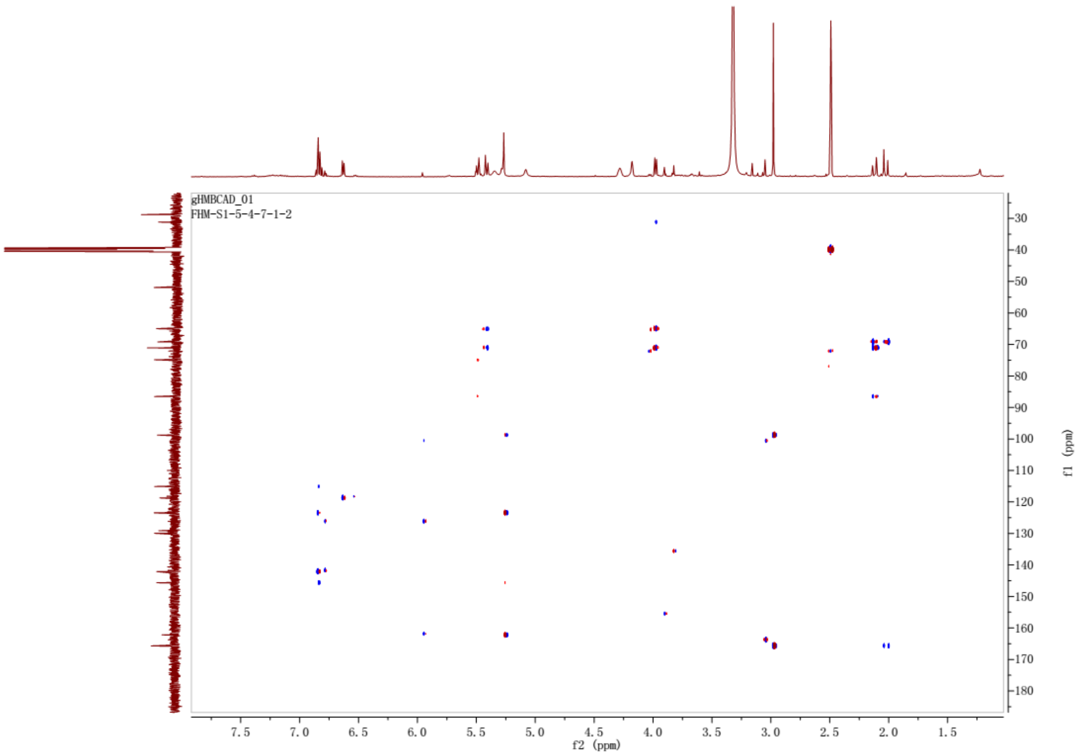

Figure S28. HMBC spectrum of penicisulfuranol J (**4**) in *d*6-DMSO

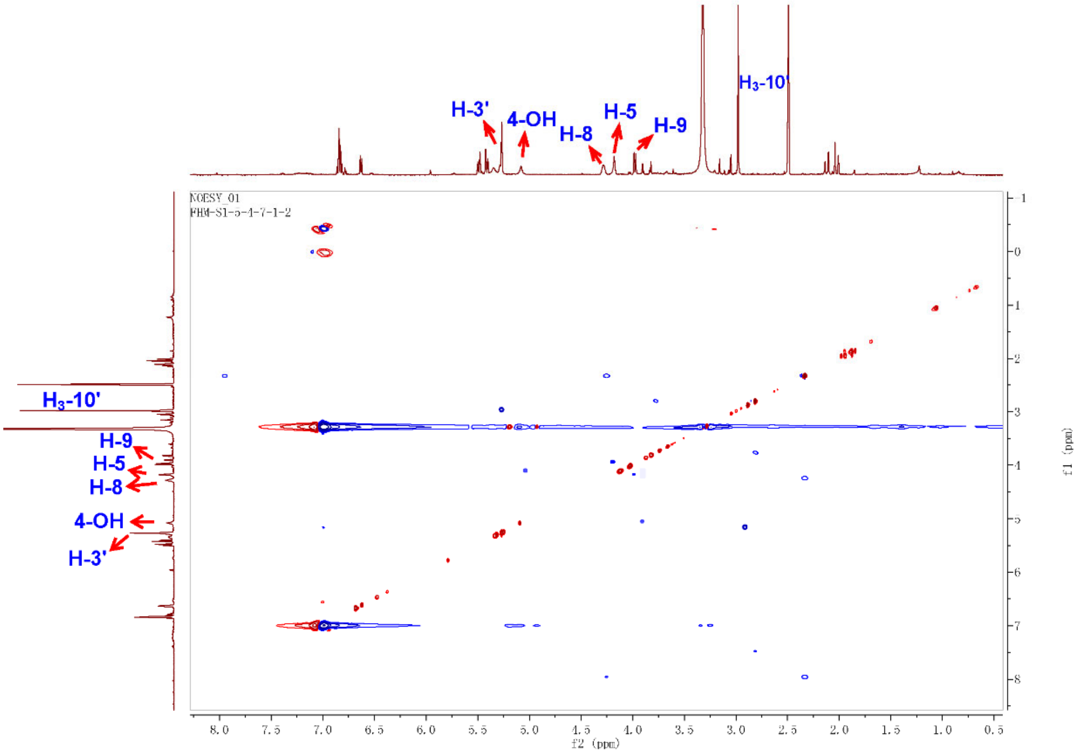

Figure S29. NOESY spectrum of penicisulfuranol J (**4**) in d_6-DMSO

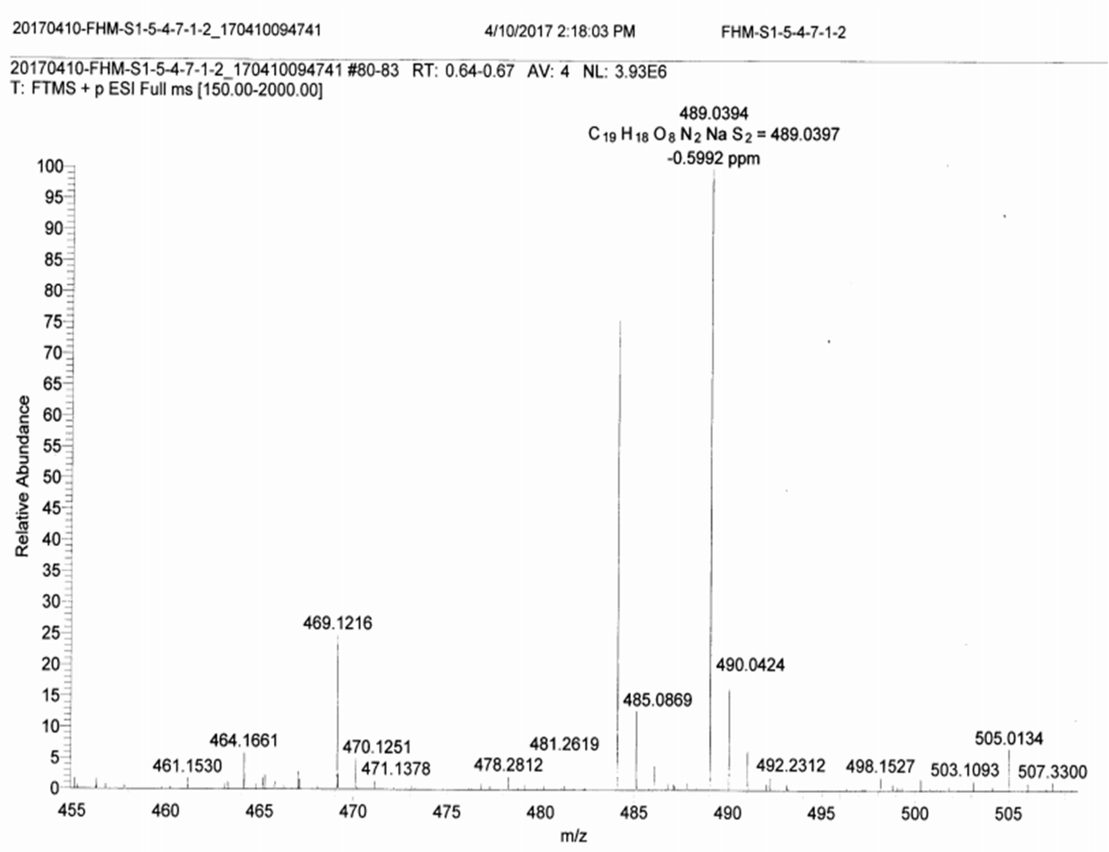

Figure S30. HRESIMS spectrum of penicisulfuranol J (**4**)

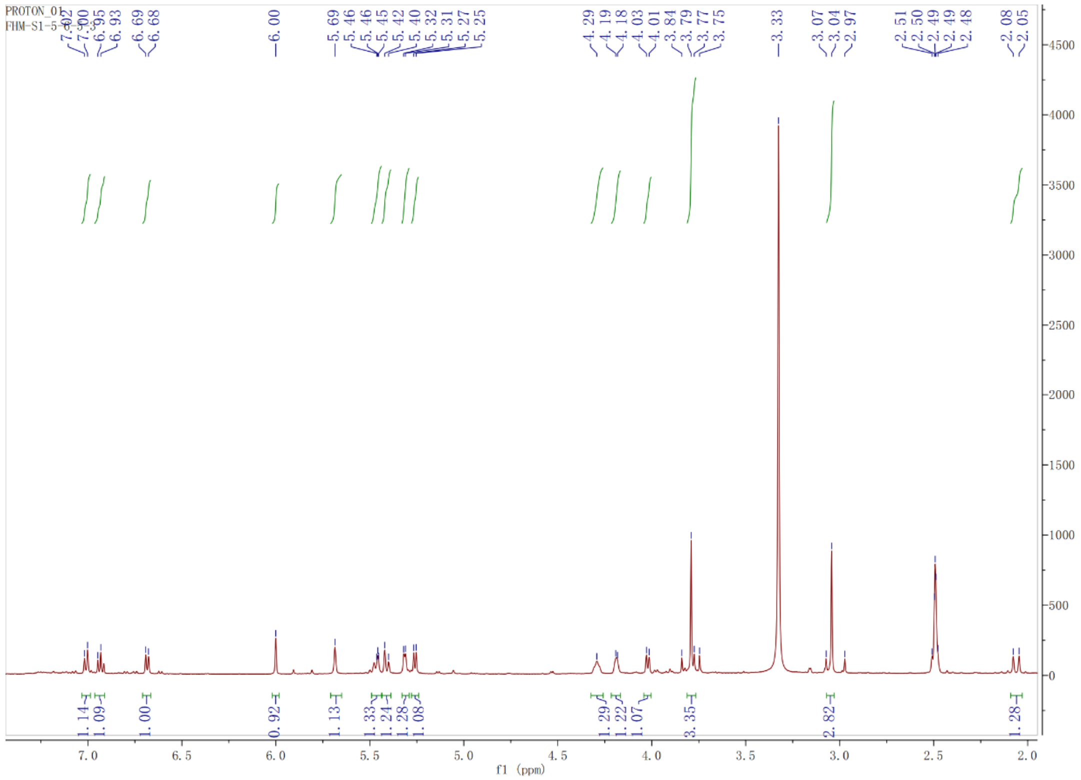

Figure S31. ^{1}H NMR (500 MHz) spectrum of penicisulfuranol K (**5**) in d_6-DMSO

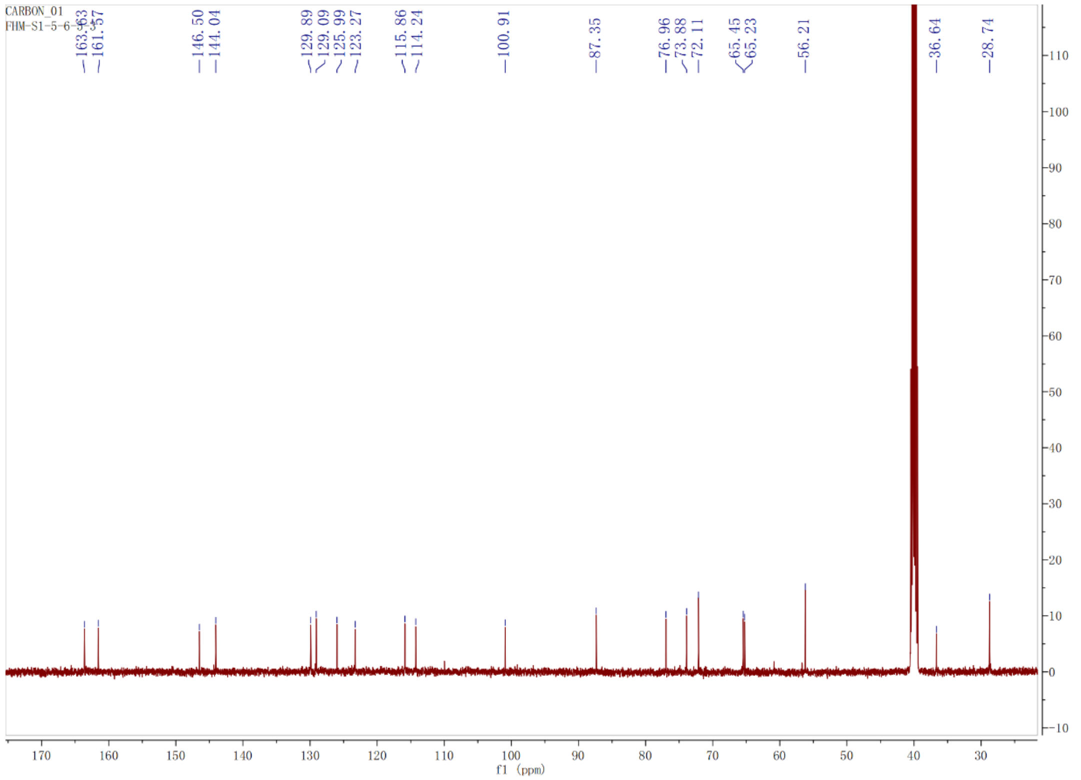

Figure S32. ^{13}C NMR (125 MHz) spectrum of penicisulfuranol K (**5**) in d_6-DMSO

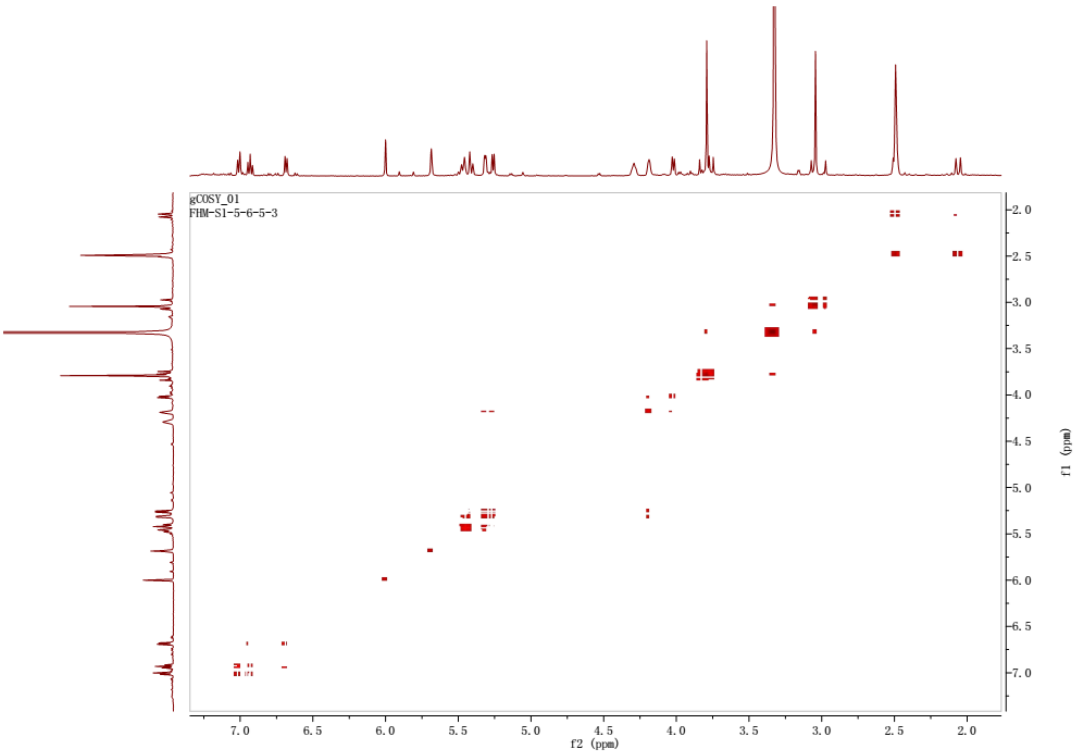

Figure S33. ^{1}H-^{1}H COSY spectrum of penicisulfuranol K (**5**) in d_6-DMSO

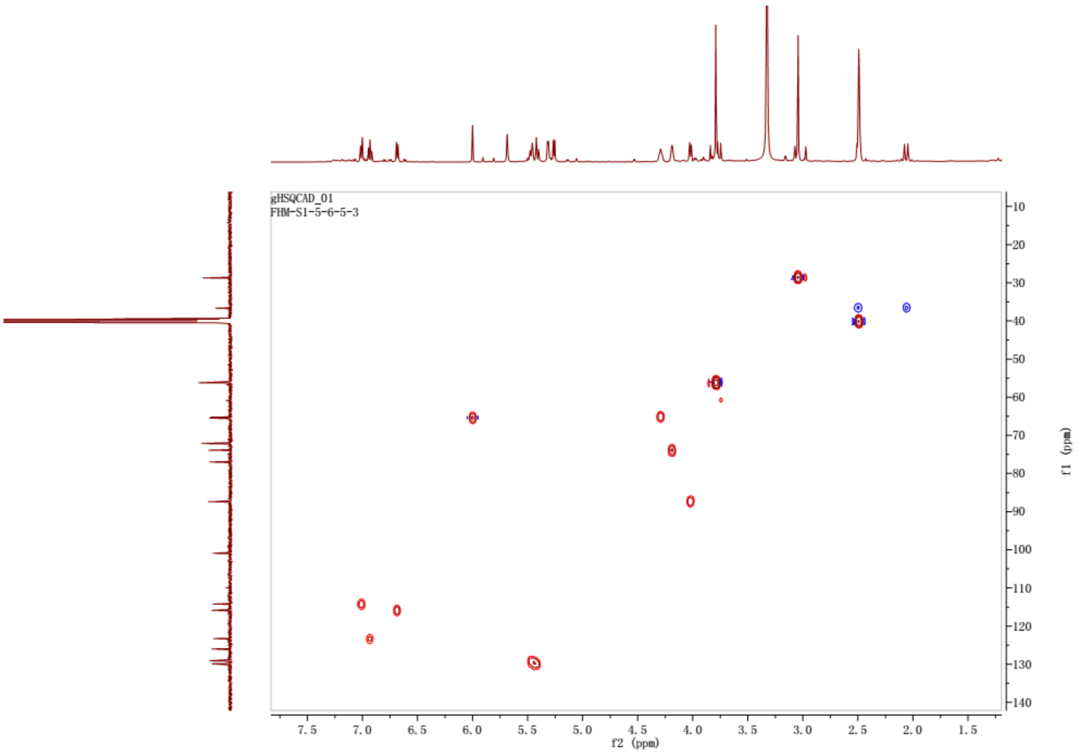

Figure S34. HSQC spectrum of penicisulfuranol K (**5**) in d_6-DMSO

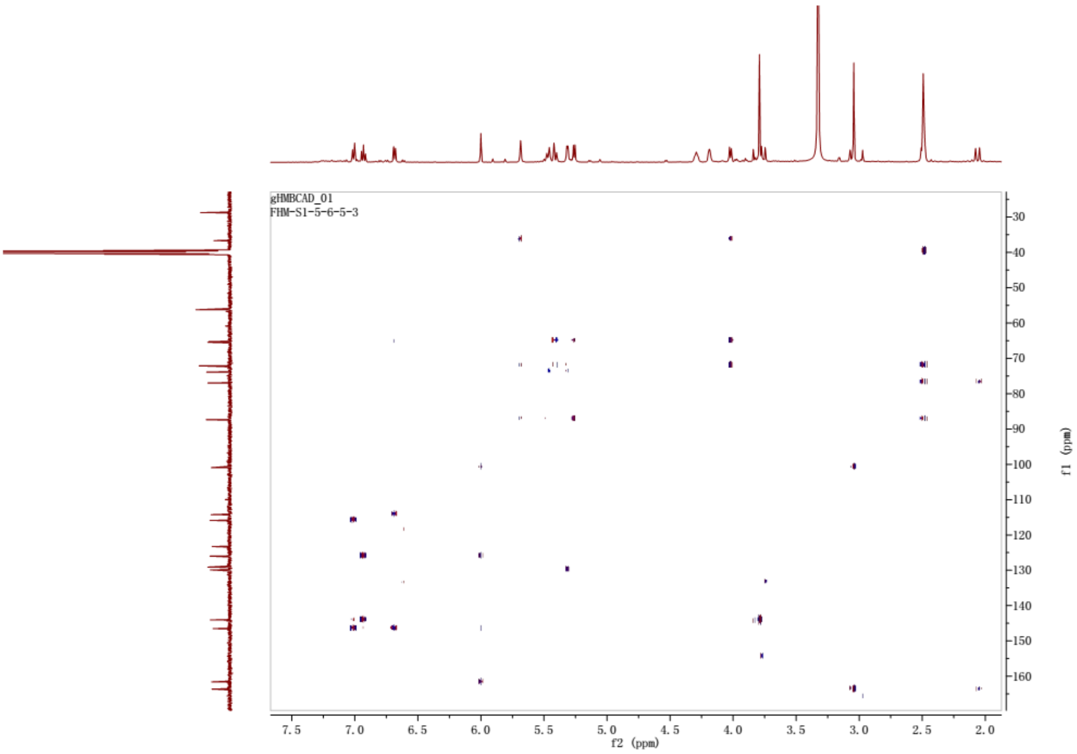

Figure S35. HMBC spectrum of penicisulfuranol K (**5**) in d_6-DMSO

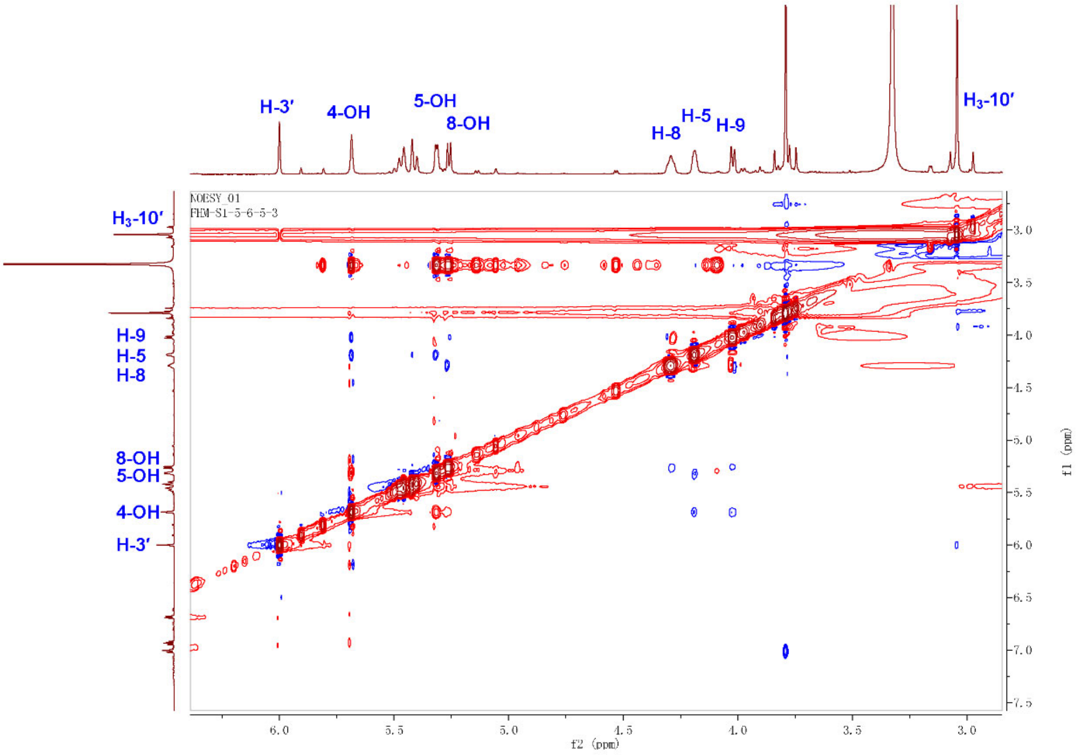

Figure S36. NOESY spectrum of penicisulfuranol K (**5**) in d_6-DMSO

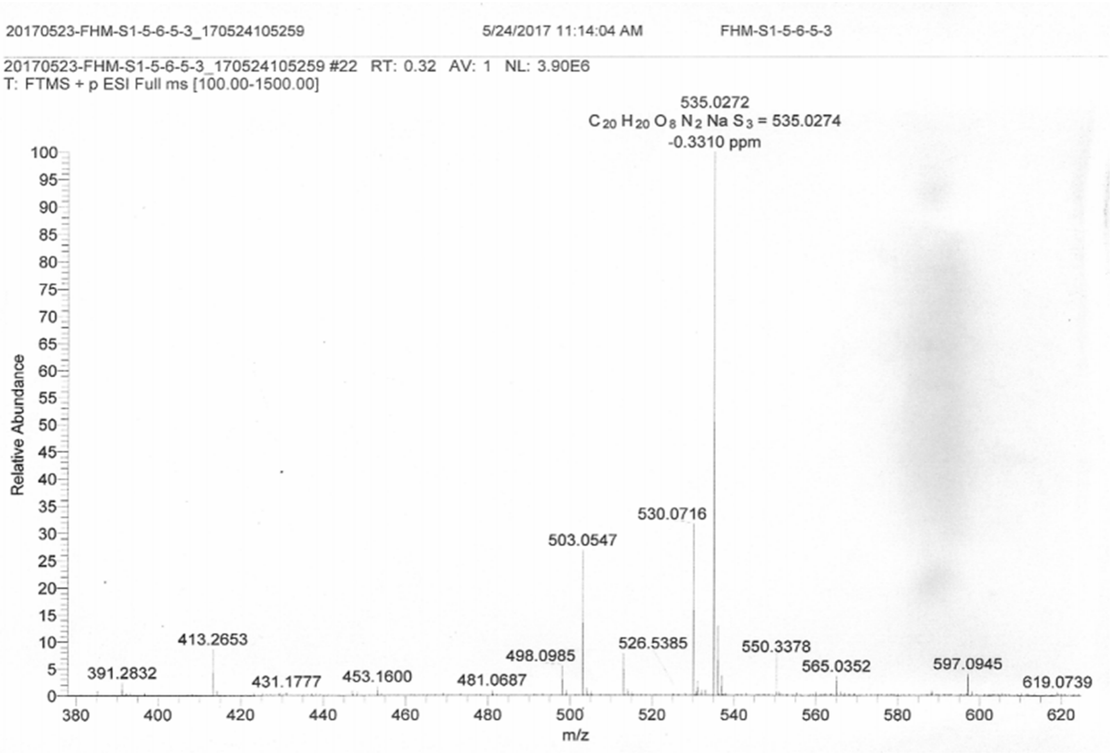

Figure S37. HRESIMS spectrum of penicisulfuranol K (**5**)

Table S1. Cartesian coordinates of the low-energy reoptimized conformers of **1** calculated at B3LYP/6-31 + G(d) level of theory with PCM solvent model for MeOH.

Conformer **1a**		Standard Orientation (Ångstroms)		
number	Atom	X	Y	Z
1	C	3.30095	1.89579	1.22491
2	C	2.97349	2.58821	-0.0748
3	C	3.68036	1.92532	-1.26413
4	C	3.56993	0.39952	-1.24452
5	C	4.03133	-0.23787	0.09325
6	C	3.75607	0.64083	1.29579
7	O	2.2388	-0.04882	-1.63406
8	N	1.4432	-0.34467	-0.52783
9	C	1.83124	-1.50519	0.22005
10	C	3.3653	-1.62252	0.21327
11	C	0.17755	0.16886	-0.52124
12	N	0.01547	-0.82384	1.75679
13	C	1.24167	-1.4135	1.65116
14	O	5.44363	-0.4955	0.02312
15	O	5.0912	2.18086	-1.21849
16	O	1.85723	-1.88496	2.60284

17	O	-0.21349	1.07488	-1.23678
18	C	-0.70274	-0.859	3.03972
19	C	-0.74349	-0.5028	0.55094
20	O	3.43561	3.95025	-0.08043
21	O	-1.74422	0.45597	0.8833
22	C	-2.89887	0.11526	0.21052
23	C	-2.84352	-1.13929	-0.39408
24	C	-1.50966	-1.7421	-0.05303
25	C	-4.0272	0.93157	0.16006
26	C	-5.14654	0.41989	-0.53342
27	C	-5.10288	-0.8411	-1.14326
28	C	-3.95057	-1.63241	-1.0787
29	S	1.10358	-3.15492	-0.50321
30	S	-0.59613	-2.46472	-1.4916
31	O	-4.08041	2.14805	0.79016
32	C	-3.35791	3.20837	0.12817
33	O	-6.27942	1.18453	-0.6754
34	C	-7.08281	1.33409	0.51178
35	H	3.14651	2.47143	2.13654
36	H	1.89024	2.57538	-0.25613
37	H	3.25999	2.31293	-2.20048
38	H	4.20094	-0.00258	-2.04098
39	H	3.9879	0.19091	2.25884
40	H	3.68704	-2.11995	1.13008
41	H	3.68925	-2.22389	-0.64024
42	H	5.87411	0.36474	-0.14418
43	H	5.20592	3.11337	-0.96004
44	H	-1.54848	-1.55343	2.99078
45	H	-1.06793	0.13771	3.28816
46	H	-0.0022	-1.19982	3.79954
47	H	2.93662	4.46688	0.57279
48	H	-1.58687	-2.53319	0.70022
49	H	-5.98507	-1.1876	-1.67244
50	H	-3.92923	-2.6108	-1.54946
51	H	-3.48777	4.09341	0.75324
52	H	-2.29534	2.96066	0.04506
53	H	-3.78001	3.38901	-0.86669
54	H	-7.92707	1.96332	0.22443
55	H	-6.51238	1.81645	1.31063
56	H	-7.4476	0.35539	0.8457

Conformer **1b**	Standard Orientation (Ångstroms)

number	Atom	X	Y	Z
1	C	3.29894	1.89523	1.22777
2	C	2.96774	2.58992	-0.06467
3	C	3.67145	1.92543	-1.2624
4	C	3.56991	0.39887	-1.24648
5	C	4.03077	-0.23782	0.09122
6	C	3.75219	0.63955	1.29425
7	O	2.23969	-0.05302	-1.63706
8	N	1.44417	-0.34758	-0.53065
9	C	1.83252	-1.50771	0.21761
10	C	3.3668	-1.62368	0.21041
11	C	0.17957	0.1681	-0.52214
12	N	0.01768	-0.82592	1.75518
13	C	1.24345	-1.41617	1.64893
14	O	5.44372	-0.49225	0.02353
15	O	5.07741	2.1984	-1.21558
16	O	1.8592	-1.88838	2.60014
17	O	-0.21045	1.07566	-1.23644
18	C	-0.69995	-0.86033	3.03845
19	C	-0.74152	-0.50376	0.54976
20	O	3.42282	3.95201	0.04779
21	O	-1.7419	0.45491	0.8831
22	C	-2.89684	0.115	0.21035
23	C	-2.84188	-1.13903	-0.39537
24	C	-1.5082	-1.74254	-0.05471
25	C	-4.0252	0.93146	0.16135
26	C	-5.14489	0.42036	-0.53198
27	C	-5.10159	-0.84009	-1.14299
28	C	-3.94933	-1.63154	-1.0798
29	S	1.10441	-3.15704	-0.50567
30	S	-0.59525	-2.46552	-1.49349
31	O	-4.07917	2.14702	0.79294
32	C	-3.34665	3.20619	0.14021
33	O	-6.2779	1.18506	-0.67256
34	C	-7.08077	1.33307	0.51516
35	H	3.14443	2.47524	2.1353
36	H	1.88268	2.58449	-0.23104
37	H	3.24652	2.30778	-2.20007
38	H	4.20193	-0.0016	-2.0431
39	H	3.98017	0.18763	2.25714
40	H	3.68927	-2.12122	1.1269
41	H	3.6912	-2.22408	-0.6436
42	H	5.87257	0.36909	-0.14227
43	H	5.16932	3.11675	-0.89747

44	H	-1.54822	-1.55159	2.98886
45	H	-1.06135	0.13733	3.28868
46	H	-0.0002	-1.20486	3.79731
47	H	2.92682	4.50451	-0.57935
48	H	-1.58576	-2.53371	0.69843
49	H	-5.98413	-1.18602	-1.67197
50	H	-3.92835	-2.60959	-1.55129
51	H	-3.759	3.39065	-0.85806
52	H	-3.47922	4.09016	0.76619
53	H	-2.28425	2.95501	0.06641
54	H	-7.92538	1.96235	0.22893
55	H	-6.51013	1.81475	1.31427
56	H	-7.44508	0.35389	0.84821

Conformer **1c**		Standard Orientation (Ångstroms)		
number	Atom	X	Y	Z
1	C	3.28765	1.96783	1.18285
2	C	2.92861	2.6547	-0.11145
3	C	3.63314	2.0086	-1.31132
4	C	3.55766	0.48071	-1.29145
5	C	4.05358	-0.14657	0.03861
6	C	3.773	0.72379	1.24607
7	O	2.23112	0.00233	-1.66072
8	N	1.46032	-0.31394	-0.54256
9	C	1.88721	-1.46659	0.19613
10	C	3.42334	-1.54732	0.16539
11	C	0.18361	0.1709	-0.51406
12	N	0.08057	-0.83213	1.76321
13	C	1.31822	-1.39272	1.63641
14	O	5.47063	-0.36903	-0.05174
15	O	5.03838	2.29619	-1.28571
16	O	1.95935	-1.85277	2.5768
17	O	-0.23898	1.06904	-1.22154
18	C	-0.61498	-0.88689	3.0578
19	C	-0.70491	-0.5247	0.5708
20	O	3.36226	4.02612	-0.12075
21	O	-1.72172	0.40929	0.92291
22	C	-2.87578	0.04907	0.26052
23	C	-2.80188	-1.20318	-0.34862
24	C	-1.45143	-1.77896	-0.02653
25	C	-4.02376	0.83685	0.23299
26	C	-5.12955	0.31804	-0.4786

27	C	-5.06977	-0.94253	-1.08611
28	C	-3.90661	-1.71839	-1.01965
29	S	1.18667	-3.13146	-0.51953
30	S	-0.54303	-2.47799	-1.48008
31	O	-4.03801	2.07537	0.82158
32	C	-4.75991	2.1506	2.06929
33	O	-6.30681	1.02639	-0.53417
34	C	-6.29356	2.18359	-1.3949
35	H	3.13017	2.53834	2.09722
36	H	1.84358	2.6195	-0.2784
37	H	3.19071	2.38725	-2.24117
38	H	4.18539	0.09341	-2.09779
39	H	4.02709	0.27811	2.20548
40	H	3.77113	-2.03871	1.07592
41	H	3.74859	-2.13878	-0.69453
42	H	5.87705	0.50186	-0.2242
43	H	5.13538	3.23102	-1.02838
44	H	-1.44006	-1.60674	3.02414
45	H	-1.00545	0.09891	3.31093
46	H	0.1081	-1.20511	3.80622
47	H	2.85524	4.53326	0.53368
48	H	-1.50159	-2.57375	0.72515
49	H	-5.94778	-1.30169	-1.61386
50	H	-3.87233	-2.69756	-1.48799
51	H	-4.29977	1.49187	2.81444
52	H	-4.68378	3.18896	2.39614
53	H	-5.8102	1.87964	1.92504
54	H	-7.28809	2.62599	-1.31637
55	H	-6.10382	1.87964	-2.43095
56	H	-5.53717	2.90388	-1.06971

Conformer **1d**		Standard Orientation (Ångstroms)		
number	Atom	X	Y	Z
1	C	3.28955	1.96474	1.18773
2	C	2.91911	2.65504	-0.09633
3	C	3.61766	2.01158	-1.30858
4	C	3.55527	0.48284	-1.29599
5	C	4.05408	-0.14628	0.03178
6	C	3.77486	0.72053	1.24223
7	O	2.23094	-0.00212	-1.6663
8	N	1.46086	-0.317	-0.54757

9	C	1.88927	-1.46851	0.19193
10	C	3.42548	-1.548	0.15795
11	C	0.18488	0.16916	-0.51673
12	N	0.08598	-0.831	1.76167
13	C	1.32272	-1.39326	1.63303
14	O	5.47138	-0.36665	-0.06059
15	O	5.01686	2.32007	-1.28599
16	O	1.96489	-1.85373	2.5725
17	O	-0.23792	1.06766	-1.22378
18	C	-0.60653	-0.88354	3.05796
19	C	-0.7019	-0.52507	0.57029
20	O	3.3431	4.02742	0.01268
21	O	-1.71813	0.40932	0.92276
22	C	-2.87309	0.04844	0.26234
23	C	-2.80034	-1.20478	-0.34494
24	C	-1.44941	-1.7802	-0.02413
25	C	-4.02076	0.83665	0.23446
26	C	-5.12771	0.31683	-0.47442
27	C	-5.0691	-0.94455	-1.08028
28	C	-3.90596	-1.72054	-1.0141
29	S	1.18768	-3.13409	-0.5206
30	S	-0.54383	-2.48127	-1.4785
31	O	-4.03507	2.07665	0.81995
32	C	-4.74224	2.15013	2.0761
33	O	-6.30483	1.02558	-0.52839
34	C	-6.2941	2.17877	-1.39445
35	H	3.1363	2.53799	2.09975
36	H	1.83181	2.62416	-0.24374
37	H	3.16632	2.38562	-2.23727
38	H	4.18408	0.10113	-2.10427
39	H	4.0305	0.27161	2.19956
40	H	3.77561	-2.04016	1.06718
41	H	3.74932	-2.13809	-0.70344
42	H	5.87695	0.50535	-0.22895
43	H	5.09086	3.23966	-0.96671
44	H	-1.42606	-1.61005	3.03068
45	H	-1.00392	0.10078	3.30571
46	H	0.12058	-1.1915	3.80677
47	H	2.81959	4.57026	-0.60036
48	H	-1.49829	-2.57391	0.72877
49	H	-5.94789	-1.30452	-1.60616
50	H	-3.87247	-2.70039	-1.48109
51	H	-4.67035	3.19002	2.39902
52	H	-5.79211	1.87063	1.94516

53	H	-4.26821	1.49724	2.81767
54	H	-7.28775	2.62275	-1.31361
55	H	-6.10917	1.86996	-2.42996
56	H	-5.53547	2.89959	-1.07569

Conformer **1e**			Standard Orientation (Ångstroms)	
number	Atom	X	Y	Z
1	C	3.28551	1.89202	1.22943
2	C	2.94829	2.59005	-0.06756
3	C	3.68043	1.93608	-1.24747
4	C	3.57589	0.40609	-1.24106
5	C	4.03399	-0.23564	0.09706
6	C	3.75528	0.64172	1.29975
7	O	2.24253	-0.04412	-1.63302
8	N	1.44457	-0.34218	-0.52924
9	C	1.83197	-1.50283	0.21834
10	C	3.36558	-1.61995	0.21275
11	C	0.17852	0.17035	-0.52394
12	N	0.01528	-0.82281	1.75407
13	C	1.24127	-1.4127	1.64903
14	O	5.44545	-0.50137	0.0309
15	O	5.07646	2.26959	-1.09554
16	O	1.85581	-1.88569	2.60063
17	O	-0.21263	1.07575	-1.24031
18	C	-0.70343	-0.85709	3.03669
19	C	-0.74291	-0.5015	0.5478
20	O	3.25644	3.98407	-0.00924
21	O	-1.74394	0.45694	0.87982
22	C	-2.89872	0.11551	0.20766
23	C	-2.84286	-1.13891	-0.39713
24	C	-1.50853	-1.74096	-0.05659
25	C	-4.02788	0.93094	0.15862
26	C	-5.14756	0.41798	-0.5334
27	C	-5.1034	-0.843	-1.14331
28	C	-3.95027	-1.63317	-1.08038
29	S	1.10545	-3.15253	-0.50688
30	S	-0.59443	-2.4626	-1.49535
31	O	-4.08286	2.14695	0.78905
32	C	-3.34784	3.20546	0.13783
33	O	-6.28164	1.18112	-0.67378
34	C	-7.08312	1.33041	0.5147
35	H	3.12218	2.47073	2.13669

36	H	1.86907	2.54502	-0.25041
37	H	3.29651	2.33695	-2.19052
38	H	4.2041	0.00203	-2.03893
39	H	3.99236	0.19324	2.26232
40	H	3.68645	-2.11907	1.12891
41	H	3.69044	-2.21976	-0.64156
42	H	5.88631	0.35908	-0.10025
43	H	5.4767	2.38689	-1.97192
44	H	-1.55035	-1.55004	2.98726
45	H	-1.06689	0.14021	3.2853
46	H	-0.00371	-1.19933	3.79665
47	H	4.22626	4.05725	-0.07585
48	H	-1.58515	-2.53239	0.69636
49	H	-5.986	-1.19031	-1.67129
50	H	-3.9286	-2.6116	-1.55105
51	H	-2.28543	2.95373	0.0668
52	H	-3.75764	3.38982	-0.8615
53	H	-3.48147	4.08967	0.76324
54	H	-7.92883	1.95817	0.22838
55	H	-6.51203	1.81426	1.31217
56	H	-7.44587	0.35143	0.85004

Table S2. Cartesian coordinates of the low-energy reoptimized conformers of **4** calculated at B3LYP/6-31 + G(d) level of theory with PCM solvent model for MeOH.

Conformer **4a**		Standard Orientation (Ångstroms)		
number	Atom	X	Y	Z
1	C	3.21686	2.10454	1.11937
2	C	3.29846	2.70193	-0.07351
3	C	3.5615	1.92575	-1.33321
4	C	3.05081	0.48628	-1.24521
5	C	3.46222	-0.25222	0.04797
6	C	3.37416	0.62729	1.35799
7	O	1.61894	0.45586	-1.48755
8	N	0.86909	0.04421	-0.38183
9	C	1.11517	-1.29338	0.07161
10	C	2.63736	-1.55318	0.11676
11	C	-0.34492	0.66387	-0.25524
12	N	-0.73459	-0.87553	1.66819
13	C	0.43386	-1.53933	1.44302
14	O	4.82505	-0.66634	-0.11966
15	O	4.98449	1.77289	-1.5871

16	O	0.92045	-2.34505	2.2331
17	O	-0.62665	1.74775	-0.73572
18	C	-1.53607	-1.19262	2.8592
19	C	-1.37937	-0.16209	0.57452
20	O	-2.31344	0.76985	1.11537
21	C	-3.44332	0.7384	0.31834
22	C	-3.46769	-0.30908	-0.59518
23	C	-2.21681	-1.11489	-0.36991
24	C	-4.4798	1.65946	0.43421
25	C	-5.57764	1.49266	-0.41901
26	C	-5.61596	0.43948	-1.3409
27	C	-4.56212	-0.48021	-1.44064
28	O	-4.46086	2.69279	1.32995
29	O	2.31792	0.25014	2.24934
30	S	0.29718	-2.62031	-1.09308
31	S	-1.28658	-1.53673	-1.91259
32	H	3.0423	2.6906	2.01941
33	H	3.19752	3.78183	-0.1567
34	H	3.09568	2.41404	-2.19696
35	H	3.4723	-0.07813	-2.08248
36	H	4.3329	0.46121	1.86729
37	H	2.87246	-2.12602	1.01546
38	H	2.94121	-2.15409	-0.74432
39	H	5.3214	0.09219	-0.48836
40	H	5.42335	2.63092	-1.46129
41	H	-2.42842	-1.76697	2.58659
42	H	-0.91556	-1.79008	3.52401
43	H	-1.83812	-0.27119	3.35751
44	H	-2.41886	-2.05877	0.14739
45	H	-6.39818	2.20134	-0.35313
46	H	-6.48022	0.33865	-1.99058
47	H	-4.60032	-1.29698	-2.15485
48	H	-3.61697	2.69704	1.81416
49	H	2.59913	-0.51265	2.77992

Conformer **4b**		Standard Orientation (Ångstroms)		
number	Atom	X	Y	Z
1	C	3.21738	2.10423	1.12011
2	C	3.29883	2.70201	-0.07259
3	C	3.56125	1.92625	-1.33266
4	C	3.05064	0.48672	-1.24509
5	C	3.46222	-0.25219	0.04789

6	C	3.37414	0.62683	1.35818
7	O	1.61885	0.45604	-1.48745
8	N	0.86911	0.04424	-0.38166
9	C	1.11526	-1.29339	0.07142
10	C	2.63744	-1.55324	0.11627
11	C	-0.34504	0.66378	-0.25501
12	N	-0.7346	-0.87622	1.66798
13	C	0.43397	-1.5398	1.44271
14	O	4.82507	-0.66614	-0.11993
15	O	4.98419	1.77352	-1.5872
16	O	0.92065	-2.34563	2.23265
17	O	-0.6268	1.74772	-0.73525
18	C	-1.53622	-1.19368	2.85885
19	C	-1.37944	-0.16245	0.5746
20	O	-2.31349	0.76933	1.1158
21	C	-3.44328	0.73832	0.31863
22	C	-3.46771	-0.30887	-0.59523
23	C	-2.2169	-1.1149	-0.37013
24	C	-4.47961	1.65953	0.43461
25	C	-5.57737	1.49316	-0.41884
26	C	-5.61573	0.44028	-1.34105
27	C	-4.56201	-0.47955	-1.44091
28	O	-4.46062	2.69263	1.33058
29	O	2.31757	0.24973	2.24918
30	S	0.29702	-2.62021	-1.09366
31	S	-1.28659	-1.53629	-1.91285
32	H	3.04331	2.69004	2.02041
33	H	3.19828	3.78198	-0.15535
34	H	3.09513	2.41488	-2.19605
35	H	3.47227	-0.07732	-2.08255
36	H	4.33271	0.46031	1.86765
37	H	2.87265	-2.12638	1.01476
38	H	2.9412	-2.1538	-0.74507
39	H	5.32131	0.09248	-0.48861
40	H	5.42293	2.63171	-1.46205
41	H	-2.42884	-1.76742	2.58584
42	H	-0.91594	-1.79188	3.52319
43	H	-1.83782	-0.27243	3.35776
44	H	-2.41909	-2.05891	0.14684
45	H	-6.39779	2.20197	-0.35287
46	H	-6.47991	0.3398	-1.99089
47	H	-4.60024	-1.2961	-2.15538
48	H	-3.61692	2.69646	1.81515
49	H	2.59832	-0.51343	2.77948

Conformer **4c**		Standard Orientation (Ångstroms)		
number	Atom	X	Y	Z
1	C	3.28167	2.09882	1.13751
2	C	3.38077	2.69479	-0.05412
3	C	3.59934	1.91716	-1.31814
4	C	3.05449	0.48604	-1.22687
5	C	3.4591	-0.25731	0.06457
6	C	3.37099	0.61585	1.37599
7	O	1.61907	0.4841	-1.46194
8	N	0.86367	0.05071	-0.36833
9	C	1.10922	-1.29069	0.07365
10	C	2.63077	-1.55534	0.12677
11	C	-0.35036	0.66863	-0.23892
12	N	-0.75175	-0.89303	1.66332
13	C	0.4219	-1.54735	1.44085
14	O	4.82274	-0.67166	-0.10156
15	O	5.03382	1.83624	-1.53149
16	O	0.91065	-2.35233	2.23063
17	O	-0.63015	1.75854	-0.70745
18	C	-1.55881	-1.22022	2.84782
19	C	-1.38985	-0.16735	0.57429
20	O	-2.32752	0.75788	1.12069
21	C	-3.45227	0.7361	0.31617
22	C	-3.47104	-0.30063	-0.60968
23	C	-2.22184	-1.10946	-0.38541
24	C	-4.4894	1.65584	0.43654
25	C	-5.582	1.4992	-0.42528
26	C	-5.61466	0.45693	-1.35972
27	C	-4.56025	-0.46165	-1.46381
28	O	-4.4758	2.67845	1.34456
29	O	2.27629	0.27734	2.23769
30	S	0.29677	-2.60724	-1.1075
31	S	-1.28223	-1.51616	-1.92625
32	H	3.14473	2.6925	2.0391
33	H	3.34336	3.77831	-0.1341
34	H	3.13161	2.42265	-2.16994
35	H	3.45618	-0.09372	-2.06431
36	H	4.30754	0.40847	1.91019
37	H	2.85835	-2.12953	1.02662
38	H	2.93769	-2.1566	-0.73304
39	H	5.32143	0.08632	-0.46823
40	H	5.2055	1.67136	-2.47507

41	H	-2.46049	-1.7732	2.56275
42	H	-0.94889	-1.84179	3.5002
43	H	-1.84581	-0.30444	3.3654
44	H	-2.42765	-2.05851	0.12087
45	H	-6.40293	2.20711	-0.35607
46	H	-6.47493	0.36384	-2.01581
47	H	-4.59413	-1.26994	-2.18782
48	H	-3.63458	2.67717	1.83345
49	H	2.52769	-0.474	2.79851

Conformer **4d**		Standard Orientation (Ångstroms)		
number	Atom	X	Y	Z
1	C	4.60987	1.64311	0.90088
2	C	4.09206	2.50395	0.01742
3	C	2.83566	2.13412	-0.72215
4	C	2.93302	0.67674	-1.22019
5	C	3.55743	-0.3474	-0.21629
6	C	3.90462	0.3238	1.13608
7	O	1.63097	0.22584	-1.68546
8	N	0.83521	-0.09186	-0.58848
9	C	1.13824	-1.33951	0.02757
10	C	2.65305	-1.60617	-0.07117
11	C	-0.33987	0.5794	-0.43885
12	N	-0.5433	-0.65644	1.71044
13	C	0.57868	-1.40169	1.46552
14	O	4.83893	-0.77867	-0.68923
15	O	2.63815	3.02543	-1.82
16	O	1.09111	-2.14156	2.29798
17	O	-0.62708	1.61608	-1.01498
18	C	-1.23307	-0.84783	2.99591
19	C	-1.29916	-0.10948	0.58603
20	O	-2.17869	0.90545	1.0639
21	C	-3.37749	0.77176	0.38732
22	C	-3.48589	-0.39883	-0.35403
23	C	-2.21825	-1.17873	-0.13064
24	C	-4.39866	1.7142	0.45998
25	C	-5.57115	1.43787	-0.25392
26	C	-5.69485	0.26006	-1.00106
27	C	-4.65454	-0.67826	-1.06001
28	O	-4.29566	2.86826	1.18641
29	O	2.65935	0.50919	1.82993
30	S	0.2024	-2.81828	-0.85642

31	S	-1.43701	-1.8276	-1.67867
32	H	5.50944	1.8704	1.46647
33	H	4.53073	3.47781	-0.18574
34	H	1.98184	2.20927	-0.03778
35	H	3.52222	0.67837	-2.14053
36	H	4.54019	-0.37151	1.6956
37	H	2.96352	-2.18669	0.79973
38	H	2.83219	-2.2189	-0.95906
39	H	4.74254	-1.20888	-1.55564
40	H	1.72747	2.90214	-2.13831
41	H	-1.8954	-1.72161	2.96755
42	H	-0.47578	-1.00426	3.76265
43	H	-1.81209	0.04455	3.22516
44	H	-2.36982	-2.03797	0.53108
45	H	-6.38217	2.15958	-0.21894
46	H	-6.61584	0.07586	-1.54604
47	H	-4.7588	-1.59098	-1.63856
48	H	-3.40884	2.93351	1.58129
49	H	2.84408	0.89866	2.70004
